# Biocatalysis Under Reduced Pressure; Two‐Step, One‐Pot Amide Synthesis Using an Immobilised Transaminase/Lipase Cascade in Combination With By‐Product Removal

**DOI:** 10.1002/cbic.202500951

**Published:** 2026-03-26

**Authors:** Lisa Kennedy, Nicholas Mulholland, Andrew Gomm, Dominic J. Campopiano

**Affiliations:** ^1^ School of Chemistry University of Edinburgh Edinburgh UK; ^2^ Jealott's Hill International Research Centre Syngenta UK Bracknell UK

**Keywords:** biocatalysis, enzyme immobilisation, lipase, rotary evaporator, transaminase

## Abstract

Immobilisation of biocatalysts has expanded their applicability under non‐conventional conditions, particularly for compatibility in synthetic processes by improving stability in non‐aqueous media and enabling reusability. The combination of biocatalysts in cascade reactions is often a useful strategy with significant advantages. However, the utility of cascades can be hindered by incompatibilities between enzymes and/or substrates and long reaction times. Herein, we describe the innovative use of a rotary evaporator for performing lipase‐catalysed reactions under reduced pressure, facilitating the in situ evaporation of a volatile by‐product and shifting the reaction equilibrium towards the desired product(s). This is demonstrated with an immobilised lipase‐catalysed kinetic resolution of a chiral amine that yields products of high enantiopurity. Additionally, a two‐step, one‐pot biocatalytic cascade is developed by coupling the lipase with an immobilised, pyridoxal 5′‐phosphate (PLP)‐dependent transaminase (ATA). To address the challenges of combining these biocatalysts, we optimised the immobilisation support, solvent (organic/aqueous) and water content. Together, the enantioselective ATA/lipase cascade converts a prochiral ketone substrate to an amide with high yield and > 99% enantiomeric excess (%*ee*). This methodology demonstrates that biocatalysts can be readily combined for organic synthesis with standard laboratory apparatus and encourages a similar approach to be applied to other reactions.

## Graphical abstract

The application of biocatalytic cascades for organic synthesis can be encouraged through the use of easy‐to‐handle, immobilised enzymes and common laboratory equipment. We present a sequential biocatalytic cascade reaction that couples a transaminase (ATA) with a lipase, and achieves high conversion and enantioselectivity through by‐product removal under reduced pressure.

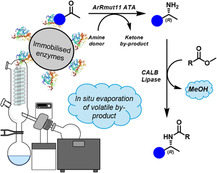

## 1 Introduction

The increasing demand for highly selective, non‐toxic and sustainable chemical processes has accelerated the adoption of biocatalysis in industry [1, 2, 3]. While a key advantage and ‘green’ feature of biocatalysts is the ability to carry out reactions in aqueous media, expanding their use beyond water is essential for broader synthetic applicability. Non‐aqueous biocatalysis can address substrate solubility limitations, simplify product recovery, and integrate enzymatic steps into established multi‐step synthetic routes conducted in organic solvents [4]. Enzyme immobilisation has significantly expanded the scope of biocatalysis in non‐aqueous media by enhancing enzyme stability, performance, and reusability [5, 6], thereby improving catalyst productivity [7, 8, 9]. Immobilised biocatalysts create opportunities for the use of a wide range of reaction conditions, including water‐immiscible organic solvents, and enable biocatalysis in continuous flow reactors [10, 11]. Additionally, it has recently been reported that artificial polyenzymes stabilised within pickering emulsions can be used to carry out complex cascade reactions [12]. This adaptability promotes wider industrial and academic adoption [5, 13] and can offer a broader range of possibilities in the engineering of synthetically useful cascade reactions, in which the product of one reaction is used as a substrate in the next [14, 15].

**FIGURE 1:**
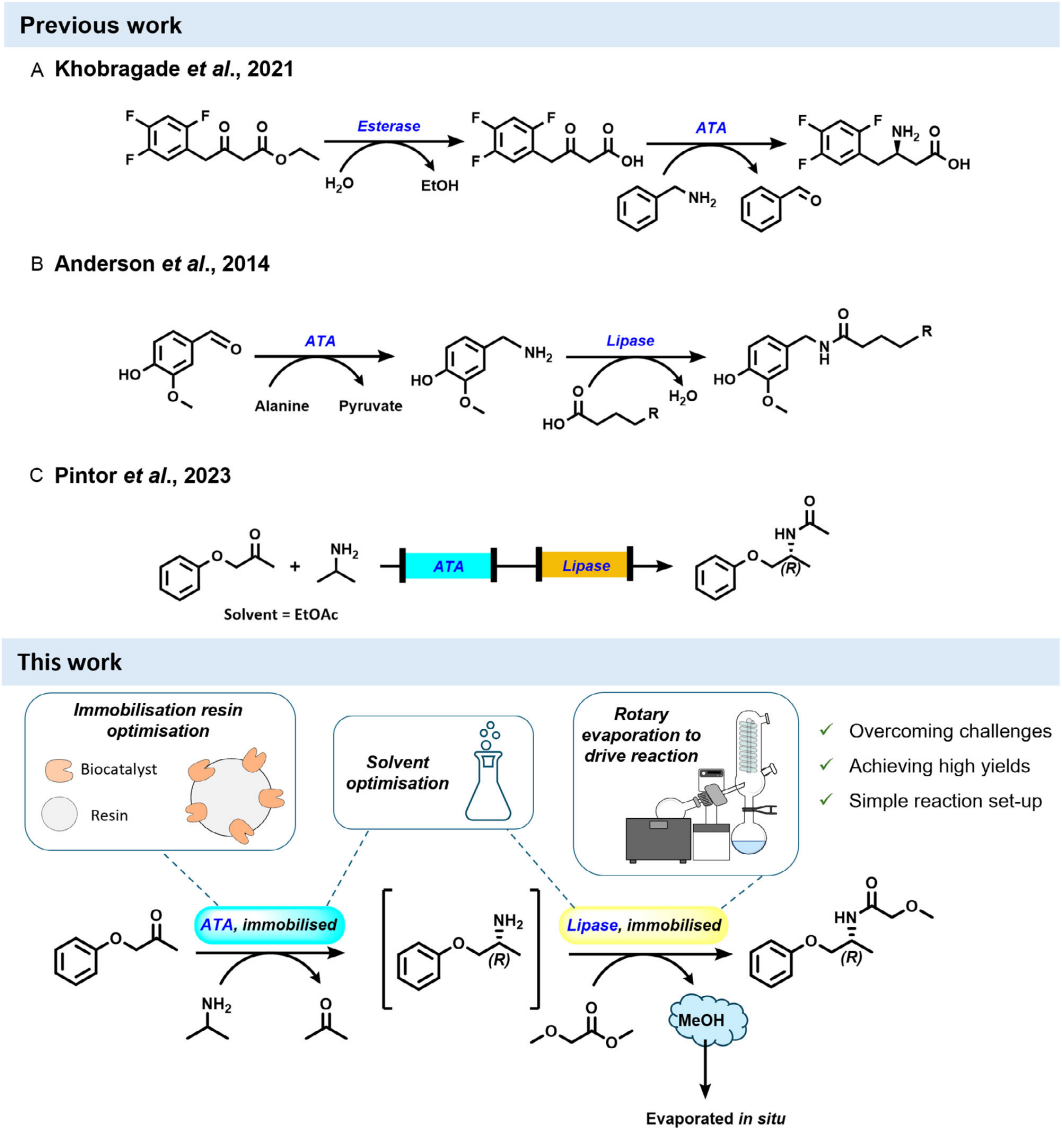
Examples of cascades involving lipase/esterase and transaminase (ATA) biocatalysts. Schemes (A–C) show previously reported work [24, 25, 26]. Scheme (A) Esterase/ATA cascade in aqueous media [24]. Scheme (B) ATA/lipase sequential cascade. The ATA step is carried out in aqueous media, while the lipase step occurs in organic solvent [25]. Scheme (C) ATA/lipase sequential cascade using immobilised enzymes in a flow reactor occurring in organic solvent [26]. The work described here – an ATA/lipase sequential cascade with immobilised enzymes in organic solvent and using a rotary evaporator to drive the lipase step under reduced pressure (200 mbar).

Amide bond formation remains a central transformation in organic synthesis, particularly for active pharmaceuticals or as key intermediates towards other target molecules [16]. There is a current demand for alternative, more sustainable methods for amide synthesis [17, 18]. Biocatalysts are already offering routes to synthetic targets, either in a single transformation [19], or when combined together in efficient cascades [14, 20]. Biocatalytic amide formation can be achieved using, for example, an amide synthetase to couple carboxylic acids and amines [21], or recently reported oxidative coupling using alcohol dehydrogenases [22]. Alternatively, amides can be prepared from acylation of the corresponding amine and transaminases (ATAs) offer a wide range of attractive routes from pro‐chiral building blocks [23]. Thus, we chose to focus on the combination of an ATA and a lipase in a one‐pot biocatalytic cascade reaction for the synthesis of chiral amides. Although both enzyme classes are widely reported individually, examples of their combined use in one‐pot cascades are still relatively limited. Within the reported examples, it is more common for a lipase/esterase and an ATA to be coupled together in that sequence, where the lipase/esterase performs a hydrolysis role and the reaction can be carried out in aqueous media. An example of such is the synthesis of a fragment of sitagliptin (Figure 1, Scheme A) [24], where the esterase hydrolyses the precursor ester to the corresponding β‐keto acid and the ATA catalyses the reductive amination of the ketone. This cascade could be conducted at a 1 g scale in a single whole‐cell system in which the esterase and ATA were co‐expressed [24].

ATAs and lipases can also be coupled in the reverse order: an ATA generates a chiral amine that is subsequently acylated by a lipase to yield an amide product [25, 26]. Successful execution of this amide‐forming cascade requires a water‐free environment for the lipase step, sequential operation to prevent competing reactions from the amine donor, and strategies to overcome equilibrium limitations. In the synthesis of capsaicin analogues (Figure 1, Scheme B), the transamination reaction was conducted in aqueous buffer before freeze‐drying the solution and resuspending in organic solvent for the lipase step [25]. This enables high yields but creates a time‐ and resource‐intensive workflow, not feasible on a large scale [25]. More recently, an ATA/lipase cascade in ethyl acetate was implemented in continuous flow using immobilised biocatalysts in separate packed bed reactors (Figure 1, Scheme C) [26]. The authors achieve a significant improvement (3.4‐fold increase in productivity) over batch reactions [26]. Continuous flow offers several advantages [27], however, these set ups are not yet widely available and can be costly to set‐up.

It is a well‐established phenomenon that the equilibrium of lipase reactions can be shifted through the removal of a short‐chained volatile by‐product using various methods [28, 29, 30]. Although few reported examples exist, Passicos et al. highlighted the use of a rotary evaporator for this purpose [31], while Sun et al. expanded on the idea in the synthesis of feruloylated monoacyl‐ and diacyl‐glycerol esters, concluding that the rotary evaporation method was more efficient at driving the reaction when compared to other strategies [32, 33]. More recently, this methodology has been applied in the scale‐up of reactions [34]. Whilst these reports focused on esterification, we report the first examples of amide formation using this strategy and the integration of this methodology within a biocatalytic cascade.

Our strategy combines the benefits of biocatalyst immobilisation with by‐product removal to improve reaction efficiency (Figure 1). As a proof of concept, we have used the kinetic resolution (KR) of a racemic amine by CALB lipase for establishing the rotary evaporator methodology. Carrying out the immobilised lipase‐catalysed reaction with an ester acyl donor under reduced pressure shifts the equilibrium by in situ evaporation of the volatile by‐product. We then showcase the synthetic utility of this combination through a sequential one‐pot, two‐step ATA/lipase reaction in which a prochiral ketone substrate is converted to an amide with high yield and >99% enantiomeric excess (%*ee*). Not only is this an effective method for driving the reaction, but it is also a simple reaction setup, taking advantage of this very common laboratory tool, which is feasible in any synthetic chemistry lab.

## 2 Results and Discussion

### 2.1 By‐Product Removal by Reduced‐Pressure Rotary Evaporation

To develop the ATA/lipase coupled cascade, we began by assessing the rotary evaporation method for the removal of the volatile by‐product generated in the lipase‐catalysed reaction. We selected the well‐established CALB‐catalysed KR of chiral amines as our model reaction because this robust biocatalyst accommodates a wide substrate scope and consistently delivers products of high enantioselectivity [35, 36, 37, 38]. In particular, CALB‐mediated resolutions employing alkyl methoxyacetates as acyl donors have been extensively demonstrated, with several studies highlighting their efficiency and versatility in resolving structurally‐diverse amines [39, 40]. This class of acyl donors was especially appropriate for developing and validating our evaporation strategy, as their aminolysis produces a small‐chain alcohol (MeOH in this case), enabling straightforward removal under reduced pressure. Using a rotary evaporator as the reaction vessel offers additional advantages, including operational simplicity, scalability, and efficient mixing of reaction components and immobilised enzymes without the need for mechanical stirring. Moreover, the by‐product alcohol can be conveniently collected and, where applicable, reused.

**FIGURE 2:**
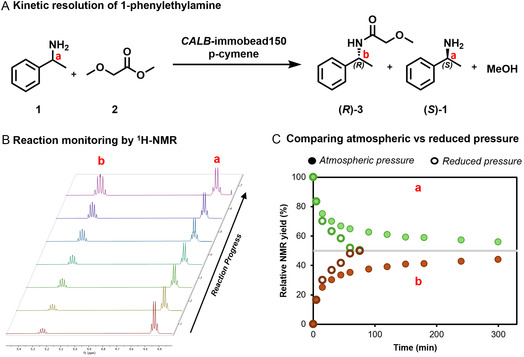
The CALB‐catalysed KR of 1‐phenylethylamine (**1**) (A). The KR of **1** by CALB in *p*‐cymene with the acyl donor (methyl methoxyacetate (**2**), and the MeOH by‐product. (B) ^1^H‐NMR reaction monitoring data for the reaction under reduced pressure (200 mbar) in the rotary evaporator. The multiplet at 4.45 ppm (**label a**) corresponds to the proton at the chiral centre of the amine (**1**), and the multiplet at 5.25 ppm (**label b**) corresponds to the proton at the chiral centre of the amide (**3**). The reaction was monitored from 5 to 80 min, and a clear change in the **a/b** ratio was observed. (C) Plotted data for the KR at atmospheric pressure (filled circles) and under reduced pressure (rings) from 0 to 300 min, showing reaction progress towards the 50% completion mark for a KR. The green circles/rings represent the relative NMR yield of **1** (based on integral of multiplet, **a**) while the maroon circles/rings represent the yield of **3** (based on integral of multiplet, **b**). Reaction conditions: 1‐phenylethylamine (7mmol), methyl methoxyacetate (MMAc, 2 eq., 14 mmol), CALB‐immobead150 (250 mg, >2000 enzyme units/g), *p*‐cymene (5 mL). The reaction was placed on the rotary evaporator at either 200 mbar or atmospheric pressure, 40°C water bath, 100 rpm.

We chose the KR of 1‐phenylethylamine (**1**) as our model substrate with methyl methoxyacetate (**2**) as the acyl donor. This reaction is used in an industrial‐scale process by BASF in the MTBE (methyl *tert*‐butyl ether) solvent [41, 42]. Due to the high enantioselectivity of CALB, the (*R*)‐enantiomer of the racemic amine is selectively acylated, and methanol is produced through the aminolysis of the acyl donor (Figure 2A). By exploiting the continuous evaporation of the methanol by‐product in the rotary evaporator (at 200 mbar pressure), we aimed to drive the equilibrium of the reaction towards the enantiopure products. The commercially available CALB employed in these reactions is immobilised on immobead150 resin. In contrast to the BASF process, a bio‐based green solvent, *p*‐cymene, was chosen as the solvent for the KR as it has a high boiling point (177 °C) and therefore, would not be easily evaporated under a reduced pressure of 200 mbar. The *p*‐cymene solvent has demonstrated high compatibility with acylation reactions, with Paggiola et al. reporting higher efficiency compared to a range of other solvents in a CALB‐catalysed ester formation [43]. It has been proposed that solvent hydrophobicity is a key factor contributing to reaction efficiency. Additionally, the absence of hydrogen bond accepting capability of the solvent molecules reduces solvent–solvent interactions, which allows the solutes to diffuse easily in and out of the active site [43]. We suggest that these features also make *p*‐cymene a useful solvent for this methodology, as its limited interaction with methanol is unlikely to affect the evaporation rate of this by‐product.

The reaction was monitored by ^1^H‐nuclear magnetic resonance (NMR) using the integral ratio between a characteristic signal of **1** and **3** (labelled a and b, respectively, Figure 2B) to determine reaction progress. A ratio of 1:1 between the signals represents KR completion as 50% of the racemic amine has been used up. Reaction completion was observed within 80 min under a reduced pressure of 200 mbar, while in comparison, the reaction under atmospheric pressure had not reached the optimal 50% yield in 5 h (Figure 2C).

Several reports of CALB‐catalysed amine KRs involve the addition of a drying reagent, such as molecular sieves, to trap the short‐chain alcohol and drive the reaction [44, 45, 46]. Nevertheless, they still require several hours to reach completion (e.g., 6 – 19 h). For example, the dynamic KR of amine‐1 required 17 h at 70°C with 30 mol% molecular sieves to reach high yields [45], while the KR of 1 in 1,2‐dimethoxyethane, employing two drying agents, required 19 h to reach completion [44]. Our proof‐of‐concept study confirmed that the rotary evaporator method was an effective way of shifting the equilibrium and achieving high yields in short reaction times. The enantiopure products, (*R*)‐2‐methoxy‐N‐(1‐phenylethyl)acetamide (***R*‐3**) and (*S*)‐1‐phenylethylamine (***S*‐1**), were isolated in 40% yield (97% *ee*) and 32% yield (99% *ee*) (ideal 50% yield for KR), respectively. To the best of our knowledge, this is the first demonstration of a CALB‐catalysed amine KR driven to completion under reduced pressure in a rotary evaporator.

### 2.2 Transaminase/Lipase Sequential Cascade

With the rotary evaporation/by‐product removal methodology established, we turned our attention to developing the more complex ATA/lipase cascade reaction. The lipase‐mediated acylation of the amine requires a water‐free environment to avoid competing ester hydrolysis. Therefore, we immobilised the ATA biocatalyst to improve stability and reusability in an organic solvent. In addition, the cascade must be carried out sequentially to prevent the amine donor from reacting in the lipase acylation and forming a major, unwanted amide side product. These criteria imposed practical considerations when constructing an efficient, one‐pot cascade reaction, including choices of compatible biocatalysts. A robust ATA was chosen and the (*R*)‐selective *Arthrobacter* sp. round 11 variant transaminase (ArRmut11) reported by Savile et al. was preferred since it was evolved/engineered to retain high catalytic activity in high % organic solvent [19].

### 2.3 Optimisation of the ArRmut11 ATA Step

To lead on from our initial work with CALB, we proposed a coupled reaction in which acetophenone (4) would be the initial substrate and form intermediate (*R*)‐1‐phenylethylamine (1) that would be a substrate for CALB to form the chiral amide, 3, isolated in the KR described above (Figure 2). Unfortunately, for this initial target amide (3), the transamination of acetophenone (4) as ketone acceptor achieved poor conversions that would limit the overall yield of the coupled reaction. To overcome this, we investigated the use of different ketone substrates in the hope that the ATA, ArRmut11, would catalyse high conversions to the intermediate amine. Docking studies suggested that slightly longer‐chained ketones are better accommodated in the ArRmut11 active site. One potential reason for this is the pi–pi stacking interaction with Tyr61 residue which may have an important role in binding the aromatic rings of the substrates and holding the ketone in a favourable orientation for reaction (Figure S3). This residue (L61Y) was incorporated in the ATA engineering/selection campaign for the transamination of pro‐sitagliptin [19]. Based on the docking results and previous literature, we tested two larger ketones, 4‐phenyl‐2‐butanone (5) and phenoxy‐2‐propanone (6) [19, 47, 48, 49].

**FIGURE 3:**
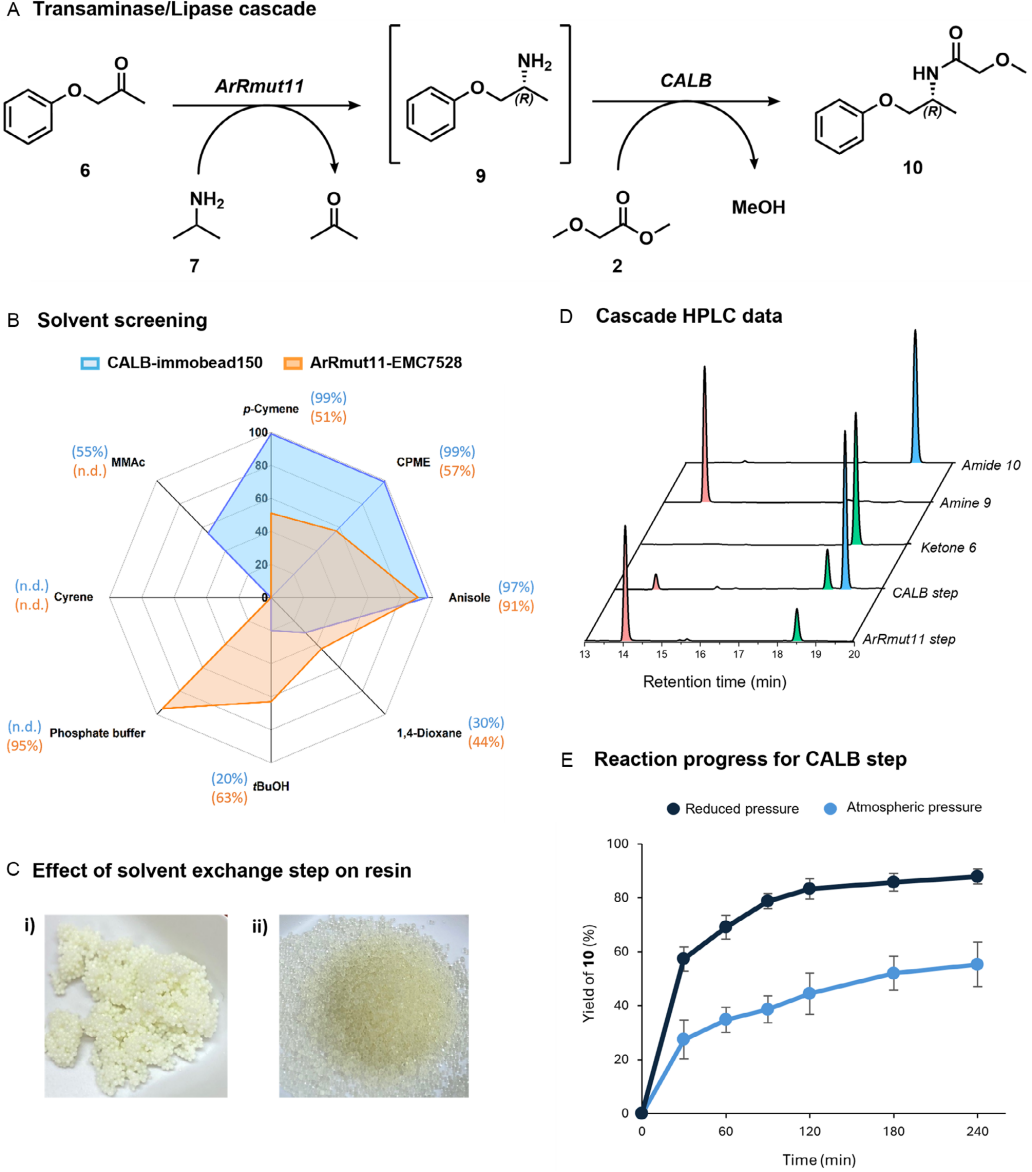
(A) Reaction scheme for the sequential biocatalytic cascade. (B) Conversion to product in various solvents for the individual reactions of ArRmut11‐EMC7528 (orange) and CALB‐immobead150 (blue). (C) Effect of solvent exchange washing step on the physical appearance of the EMC7528 resin, i) before solvent exchange, ii) after solvent exchange and drying. (D) HPLC chromatograms corresponding to the end point of each step of the preparative scale sequential cascade (immobilised ArRmut11 step and immobilised CALB step) and standards for substrate **6** (R_t_ = 18.4 min, shaded green), intermediate **9** (R_t_ = 14.2 min, shaded pink) and product **10** (R_t_ = 18.9 min, shaded blue). (E) Reaction progress for the CALB reaction of the cascade under reduced pressure (200 mbar, navy) and atmospheric pressure (light blue). Preparative scale reaction conditions: phenoxy‐2‐propanone (1 mmol), isopropylamine (5 mmol) in anisole (20 mL), 5%w/v ArRmut11‐EMC7528 (enzyme loading = 20 mg/g resin), 45 °C, 18 h, 100 rpm. ATA is filtered out, and 1%w/v CALB‐immobead150 (enzyme loading = >2000 U/g) and methyl methoxyacetate (7 mmol) are added to the filtrate. The flask was placed on the rotary evaporator at 200 mbar for 4 h, 40°C water bath, 100 rpm. Yields calculated by HPLC peak area calibration curve. Errors were calculated as the standard deviation of triplicate reactions.

Experimental results were in agreement with this hypothesis (Table 1). The reaction of 4 with ArRmut11 in buffer over 24 h was quite poor reaching only  20% yield (by high performance liquid chromatography (HPLC)) with 10 equivalents of isopropylamine (iPrNH_2_, 7) as the amine donor, and up to 65% with 50 equivalents of iPrNH_2_. However, the reactions of ketones (5) and (6) gave much higher yields (>90%) (Table 1). Therefore, in subsequent reactions, ketone (6) was chosen as substrate for the transamination step and this would result in the *R*‐enantiomer of intermediate amine (9, Figure 3A). The versatile CALB biocatalyst is capable of acylating the phenoxy‐2‐propanamine intermediate (9) [26], resulting in the target amide of the two step, one pot reaction (10, Figure 3A). Additionally, we compared two different amine donors for the ATA step, iPrNH_2_ (7) and the ‘smart’ N‐phenylputresine (NPP, 8) that offer different advantages in driving transamination reactions [50]. The iPrNH_2_ results in acetone formation which can be removed under reduced pressure. In contrast, NPP drives the reaction by forming an insoluble NPP dimer that precipitates out of the reaction [50]. The ArRmut11 was shown to work well with both amine donors for the transamination of ketone 6; with the iPrNH_2_ (2.5 equivalents) donor the reaction was essentially complete in aqueous buffer (∼99%, Table 1). Similarly, NPP (2 equivalents) under the same conditions also resulted in very high conversion to the target *R*‐amine product (98%, Table 1) [50].

**TABLE 1:** Ketone substrate (black box, 4‐6) and amine donor (grey box, 7‐8) screening for transamination catalysed by ArRmut11. Reaction conditions: Ketone (10 mM), amine donor (equivalents detailed in table), ArRmut11 (1 mg/mL) in sodium phosphate buffer (50 mM, pH 8), 20% DMSO, 24 h, 40°C. Conversion of substrate quantified by HPLC peak area.

Reaction scheme	Ketone substrate	Amine donor	Conversion, %
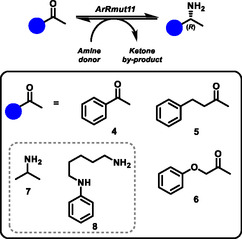	**4**	**7** (10 eq.)	20
	**4**	**7** (50 eq.)	65
	**5**	**7** (50 eq.)	92
	**6**	**7** (50 eq.)	99
	**6**	**7** (2.5 eq.)	99
	**6**	**8** (2 eq.)	98

### 2.4 Enzyme Immobilisation Screening

To facilitate the coupled reaction in an organic solvent, our strategy required immobilisation of the ArRmut11 ATA to enhance its stability in a non‐aqueous environment [51, 52, 53]. Unlike CALB, which is commercially available immobilised on immobead150, the ArRmut11 ATA required in‐house immobilisation. We carried out an immobilisation resin screen using commercial resins from Purolite/SunResin to determine the ideal carrier for ArRmut11. The resins were examined on the basis of immobilisation yield and enzyme activity. Prior to experimental resin screening, a surface residue analysis was performed using CapiPy, a Python‐based GUI‐application, to assist in protein immobilisation [54]. The results of this showed that the protein surface of ArRmut11 had several hydrophobic clusters, which could potentially bind to hydrophobic immobilisation resins. The CapiPy analysis showed that the hydrophobic amino acids (Ala, Val, Leu, Ile, Pro and Phe) make up over 38% of the surface of the protein (Figure S5). The analysis also showed the presence of lysine residues on the surface of the protein (∼3% of overall surface residues), which may be useful in covalent binding (Figure S5). We tested these predictions experimentally by screening several commercial resins. The recombinant, purified ArRmut11 was immobilised as per standard protocols (see Supporting Information) with an excess of PLP (0.1 mM) in the working buffer. After immobilisation, the resins were washed with water to remove excess buffer/PLP. High immobilisation yields were obtained at a protein loading of 20 mg ArRmut11 per gram resin for all of the tested carriers (Table 2). The activity of each immobilised ArRmut11 was tested under both aqueous and non‐aqueous conditions and was quantified through the transamination of ketone, 6 using iPrNH_2_, 7 (2.5 eq.) as the amine donor over 3 h (Table 2).

**TABLE 2:** Screening details and results for the immobilisation of purified ArRmut11 on Purolite (ECR) or SunResin (EMC) carriers. Immobilisation was carried out as per standard protocols (see Supporting Information for details). In all cases, the target protein loading was 20 mg protein per g wet carrier. Activity was measured on a 1 mL scale through the transamination of phenoxy‐2‐propanone 6 (20 mM) with isopropylamine 7 (50 mM) as amine donor after 3 h. Each resin was evaluated in aqueous media (sodium phosphate buffer 50 mM, pH 8) and organic media (anisole). Biocatalyst loading was calculated depending on the immobilisation yield to give a final protein loading of 1 mg/mL in the reaction. Before reaction in anisole, the resin was solvent exchanged and dried. Reactions were carried out in triplicate, and the error was calculated as the standard deviation. Activity refers to the reaction yield. The reactions were analysed by HPLC analysis and quantified through calibration of the ketone substrate.

Carrier specifications				Experimental specifications			
**Carrier**	** Type**	** Pore diameter, Å**	** Particle size, µm**	** Immobilisation**	** Immobilisation yield, %**	** Activity in aqueous media, %**	** Activity in organic media, %**
**ECR8285**	Epoxy	400–600	300–700	Covalent	99	98 ± 0.5	36 ± 2.3
**ECR8204**	Epoxy	300–600	150–300	Covalent	99	97 ± 1.5	21 ± 0.6
**ECR8806**	Octadecyl	500–700	150–300	Adsorption	98	95 ± 1.9	32 ± 2.2
**EMC7014**	Epoxy	400–600	150–350	Covalent	97	93 ± 0.8	29 ± 0.5
**EMC7528**	Octadecyl	200–400	400–1000	Adsorption	99	97 ± 0.2	41 ± 3.0
**Chelex7350**	Iminodiacetic	800–1000	100–250	Metal affinity	87	95 ± 1.4	5 ± 2.8

Catalytic activity was observed for all of the tested resins with comparable activity to the free enzyme in the aqueous assays. Although the quantified reaction yields in organic media were lower overall, importantly, some degree of activity was retained for all of the tested resins. We noted that the hydrophobic‐adsorbent resins performed well in the organic solvent. Similarly, the epoxy resins gave comparable yields, while the nickel affinity interaction resin (Chelex7350) was the only support which led to a significant drop in activity when used in organic media. This may be due to the nature with which the immobilisation occurs as the protein interacts via the ArRmut11 N‐terminal poly‐histidine affinity tag chelating with a Ni^2+^ ion bound to the resin, resulting in a single–point interaction, while multipoint interactions may provide enhanced stability [55]. Additionally, in organic solvents, the metal–affinity interaction could be weakened, and leaching of the nickel ion from the resin may be a factor. It was concluded that the adsorbent resin, EMC7528, would be taken forward for immobilising ArRmut11 in all further experiments due to its availability, good activity in organic solvent and high immobilisation yield. It should also be acknowledged that at + 4 °C, these immobilised biocatalyst preparations display months‐long storage stability. It was also shown that the ArRmut11 immobilised on EMC7528 could be reused up to 8 consecutive times without a significant decrease in activity (Figure S11).

### 2.5 Solvent Screening

For the cascade to be suitable as a one‐pot system without an extraction/purification step in between the biocatalytic steps, we screened various solvents to determine the most compatible with both biocatalysts. The solvents screened all have high boiling points to avoid evaporation in the rotary evaporator at 200 mbar. Although *p*‐cymene presented high compatibility with CALB in our initial method development, in contrast, it has been reported that immobilised transaminase reactions perform well in polar aprotic solvents such as esters, ethers, or DMSO [56, 57]. Before screening organic solvents, the immobilised ArRmut11‐EMC7528 was washed with a solution of isopropyl alcohol/PEG‐400/water in a 85:10:5 ratio to remove excess water from the resin (Figure 3C) [58]. If this step was omitted, the activity diminished, especially in water‐immiscible solvents. Carrying out this solvent exchange step prevents the formation of a biphasic reaction mixture in these solvents, which could otherwise hinder substrate accessibility to the enzyme. This washing step was carried out throughout this work before using the immobilised ATA. Various solvents were screened for the individual reactions catalysed by CALB‐immobead150 and ArRmut11‐EMC7528. CALB‐immobead150 was highly active in *p*‐cymene, anisole and cyclopentyl methyl ether (CPME) (Figure 3B), while no amide formation was observed in reactions with cyrene or aqueous buffer as solvent. ArRmut11‐EMC7528 performed well in aqueous buffer, anisole and CPME (Figure 3B) while methyl methoxyacetate (MMAc) and cyrene gave the poorest results. Ether solvents, such as anisole or CPME, are often suitable for biocatalysis as they are thought to be less likely to strip essential structural water molecules from the enzyme, but still provide some structural similarity (R‐O‐R) to an aqueous environment (H‐O‐H) [53, 59, 60]. Both ArRmut11 and CALB showed high activity in anisole, which could solubilise the reaction components and has a sufficiently high boiling point (154 °C), making it a compatible solvent for use under reduced pressure (200 mbar) without being evaporated. Previous reports have often seen immobilised‐ATA reactions successfully performed in ethyl acetate, MTBE or isopropanol [56, 57] However, transformations catalysed by ArRmut11ATA in anisole have not been previously reported, nor has anisole been shown to be compatible with this particular immobilised ATA/CALB cascade.

The water content in immobilised ATA reactions is an important factor for activity and stability [26, 58] The effect of additional water being added to the reaction in anisole was also investigated (0–3 v/v%) (Figure S10). Considering that the solvent is not dry and that the ArRmut11‐EMC7528 resin is washed with 5% water before use, high conversion in the transamination of ketone **6** was achieved in ’neat’ anisole without additional water. The reaction yield is not impacted by the addition of small amounts of water (up to 2% v/v), but decreased at higher levels, consistent with observations made when the solvent exchange step was omitted and a water layer remained around the resin, resulting in a biphasic system. In contrast, the addition of water to the CALB step has a significant effect on the amide yield as the lipase‐catalysed aminolysis step is hindered by the presence of water (Figure S10). Fortunately, the solvent did not need to be completely anhydrous to achieve high yields in the CALB step and the cascade reaction could be carried out without the requirement of adjusting the water content of the anisole solvent.

The immobilised ATA reaction in anisole was tested using either iPrNH_2_ (**7**) or NPP (**8**) as amine donor. The analytical scale reaction of **6** with iPrNH_2_ (5 eq.) as amine donor performed better than NPP (2 eq.) under these conditions after 22 h (90% vs. 74% conversion of ketone, respectively). We hypothesise that the decrease in reaction yield that we observe for the reaction with NPP in organic versus aqueous media (74% vs. 98%) may be due to increased solubility of the NPP dimer precipitate in organic media than in water, limiting the thermodynamic driving force. Although precipitation of the NPP dimer was observed during scale‐up (10 mL), the solid was difficult to separate from the immobilised enzyme and compromised catalyst reusability. Therefore, the cascade was developed using phenoxy‐2‐propanone (**6**) as substrate, isopropylamine (**7**) as amine donor (5 eq.) and anisole as solvent (Figure 3A). Control experiments confirmed that background imine formation between **6** and iPrNH_2_ was negligible under these conditions.

### 2.6 Preparative Scale One‐Pot, Two‐Step ATA/Lipase Reaction

Optimisation of the cascade reaction parameters allowed the sequential cascade to be carried out at a preparative scale (1 mmol) in a round‐bottom flask, making use of the rotary evaporator to drive the lipase step. Following the formation of phenoxy‐2‐propanamine (**9**) in the ATA reaction (≈80% yield), CALB‐catalysed acylation under reduced pressure was evaluated at this scale. The reaction under reduced pressure begins to plateau after 120 min at ≈90% conversion of **9**, whereas the corresponding reaction at atmospheric pressure reached only 55% conversion over the same period (Figure 3E). Notably, an amplification of enantiopurity is observed between the two steps. The ArRmut11 ATA does not display perfect enantioselectivity under these conditions and yields the (*R*)‐**9** with 85%*ee*. However, coupling with the CALB lipase, which exhibits excellent enantioselectivity, results in the final product, (*R*)‐2‐methoxy‐N(1‐phenoxy‐2‐propanyl)acetamide (**10**) in > 99%*ee* (Figure S14, 50% isolated yield). This is a significant advantage of coupling ATA‐mediated amine formation with lipase‐catalysed acylation.

Overall, the optimised one‐pot, sequential cascade achieved a combined conversion of 71 ± 3% over the two steps (Figure 3D), exceeding previously reported values for comparable ATA/lipase systems. To our knowledge, this work is the first report of using the rotary evaporator as a reaction vessel within a preparative‐scale ATA/lipase cascade reaction. By accounting for key process considerations, we overcame the associated challenges and established an adaptable framework that should be broadly applicable to this synthesis of a range of amides.

## 3 Conclusion

We have developed a one‐pot, two‐step ATA/lipase‐coupled reaction on a preparative scale for the synthesis of chiral amides of high enantiopurity. We have optimised the cascade by screening substrates, immobilisation resins and reaction solvents (both aqueous and organic) and identified the conditions that resulted in the highest yields. We have shown that standard laboratory apparatus, the rotary evaporator, can be used to drive lipase‐catalysed reactions. When carried out in a round‐bottom flask under reduced pressure, the removal of the volatile by‐product shifts the reaction equilibrium and leads to completion of the reaction in a shorter time. An immobilised, *R*‐selective ATA converted the prochiral ketone substrate with high yield, and acylation of the resultant amine by an *R*‐selective lipase gave an amide with > 99% enantiomeric excess. This methodology demonstrates that by careful consideration of the reaction conditions, biocatalysts can be combined together for organic synthesis, without the need for specialised reactors. This approach could be applied to various other systems to overcome challenges associated with the particular characteristics of each biocatalyst.

## Supporting Information

Additional supporting information can be found online in the Supporting Information section. The authors have cited additional references within the Supporting Information [61, 62, 63, 64, 65, 66, 67]. **Supporting Fig. S1**: A) Elution profile of the immobilised metal affinity chromatography (IMAC) during purification of ArRmut11. B) SDS gel analysis of purified fractions; M = marker, 1 = cell pellet, 2 = lysate, 3 = flow through during loading, 4 = flow through during column wash, 5‐11 = IMAC fractions. Expression conditions: LB media (1L cultures), kanamycin 30 ug/ml, IPTG 0.2 mM, 20°C overnight. Yield = 74 mg protein/1L culture. **Supporting Fig. S2**: ESI‐MS spectrum of purified ArRmut11. Calculated mass = 39726.24 ± 0.58 Da which corresponds to the predicted mass minus the N‐terminal methionine residue (39725.47 Da). **Supporting Fig. S3**: 4‐phenyl‐2‐butanone (cyan), phenoxy‐2‐propanone, 3 (peach) and acetophenone, 1 (indigo) docked in the active site of ArRmut11 (PDB code = 5FR9). PLP shown in yellow, residues Lys 188 and Tyr‐61 shown in pink. The pi‐pi stacking interaction seen with the tyrosine may be promoting better binding for the 4‐carbon chain substrates than that seen with acetophenone. This interaction holds the ketone molecule in the optimal area for reacting. **Supporting Fig. S4**: Example of NPP dimer precipitating in reactions when using NPP as amine donor for ArRmut11. **Supporting Fig. S5**: CapiPy results for ArRmut11. A). Surface residue analysis showing abundance of each amino acid residue on the exposed surface of the protein. B). Physical representation of surface exposed residues on the structure of ArRmut11 (PDB code: 5FR9), where clusters of amino acids are coloured green = hydrophobic, red = negative, blue = positive. **Supporting Fig. S6**: Phenoxy‐2‐propanone calibration curve produced by HPLC analysis of phenoxy‐2 propanone at concentrations 0 – 1.5 mM using HPLC method A (*t*_R_ = 19.5 min). Samples of each concentration were run in triplicates and the average peak area plotted. The error was determined as the standard deviation. **Supporting Fig. S7**: Phenoxy‐2‐propamine calibration curve produced by HPLC analysis of phenoxy‐2 propamine at concentrations 0–1.5 mM using HPLC method A (*t*_R_ = 14.2 min). Samples of each concentration were run in triplicates and the average peak area plotted. The error was determined as the standard deviation. The standard of phenoxy‐2‐propanamine used to make up a standard solution was synthesised by the procedure detailed below. **Supporting Fig. S8**: A) Reaction scheme for the derivitisation of phenoxy‐2‐propanone using Marfey's reagent. B) HPLC chromatograms for phenoxy‐2‐propanamine (P2P‐Amine) derivatised with Marfey's reagent as a method for resolving enantiomers. The black line represents the chromatogram for the reaction with ArRmut11, an R‐selective transaminase. The red line represents the chromatogram for the reaction with CVTA, an S‐selective transaminase, used as a standard. The (R, S)‐diastereomer produced in the derivatisation procedure elutes with tR = 12.7 min while the (S, S)‐diastereomer elutes with tR = 12.0 min. **Supporting Fig. S9**: Water content analysis for CALB on analytical scale from 0 – 3% added water in anisole. Reactions are shown from 0% (left hand side) to 3% (right hand side). The immobilised CALB can be seen to be clumping together as the % water increases and a water bubble is formed in the solvent. **Supporting Fig. S10**: Effect of added water (v/v%) on the reaction in anisole for ArRmut11‐EMC7528 and CALB‐immobead150. **Supporting Fig. S11**: Bar chart showing the reaction conversion for the transamination of phenoxy‐2 propanone (10 mM) by ArRmut11‐EMC7528 in anisole (1.5% H2O) over 8 consecutive uses of the same batch of immobilised enzyme. Reaction conditions detailed above. Error calculated as standard deviation of triplicate reactions. **Supporting Fig. S12**: Yield data for the biocatalytic cascade starting from phenoxy‐2‐propanone (6) to 2 methoxy‐N(1‐phenoxy‐2‐propanyl)acetamide (10). Reaction yield for the transamination of 6 to 9 by 24 ArRmut11‐EMC7528 is shown in peach. Reaction yield for the acylation of 9 to form 10 by CALB immobead150 is shown in blue. The overall yield for the two‐step cascade is shown in grey. The ee% for each reaction is shown in the orange dotted line. **Supporting Fig. S13**: Step by step of cascade reaction. 1) ArRmut11 step at atmospheric pressure 2) filtering immobilised ArRmut11 out of reaction 3) CALB step on rotary evaporator at 200 mbar. **Supporting Fig. S14**: Chiral HPLC chromatograms for A) the isolated amide product, 2‐Methoxy‐N‐(1‐phenoxy 2‐propanyl)acetamide, of the biocatalytic cascade, synthesised by ArRmut11 and CALB (99% ee). B) the amide product derived from chemical acylation of phenoxy‐2‐propanamine from the ArRmut11 reaction (85% ee). tR (R)‐amide = 15.1 min (blue), tR (S)‐amide = 15.5 min (peach), HPLC method D. The coupling of the two biocatalysts causes an amplification of enantiopurity. **Supporting Fig. S15**: Chiral HPLC chromatograms for A) the amide product formed in the KR of 1‐phenylethylamine by CALB, (R)‐2‐Methoxy‐N‐(1‐phenylethyl)acetamide (99.0% ee) and B) the racemic standard of 2‐Methoxy‐N‐(1‐phenylethyl)acetamide, synthesised chemically. tR (R)‐amide = 10.0 min (blue), tR (S)‐amide = 12.2 min (peach), HPLC method B. **Supporting Fig. S16**: Chiral HPLC chromatograms for A) the amine product formed in the KR of 1‐phenylethylamine by CALB, (S)‐1‐phenylethylamine (99.6% ee) and B) the racemic standard of 1 phenylethylamine, synthesised chemically. tR (R)‐amine = 7.4 min (blue), tR (S)‐amine = 8.2 min (peach), HPLC method B. **Supporting Fig. S17**: 1H‐NMR of (R)‐2‐Methoxy‐N‐(1‐phenylethyl)acetamide in CDCl3, 500 MHz. **Supporting Fig. S18**: ^13^C‐NMR of (R)‐2‐Methoxy‐N‐(1‐phenylethyl)acetamide in CDCl3, 500 MHz. **Supporting Fig. S19**: ^1^H‐NMR spectrum of (S)‐1‐phenylethylamine in CDCl3, 500 MHz. **Supporting Fig. S20**: ^13^C‐NMR of (S)‐1‐phenylethylamine in CDCl3, 500 MHz. **Supporting Fig. S21**: ^1^H‐NMR spectrum of phenoxy‐2‐propanamine formed by the transamination of phenoxy 2‐propanone by ArRmut11‐EMC7528. **Supporting Fig. S22**: ^13^C‐NMR spectrum of purified phenoxy‐2‐propanamine formed by the transamination of phenoxy‐2‐propanone by ArRmut11‐EMC7528. **Supporting Fig. S23**: ^1^H‐NMR spectrum of Marfeys Reagent. **Supporting Fig. S24**: ^13^C‐NMR spectrum of purified Marfey's Reagent. **Supporting Fig. S25**: ^1^H‐NMR spectrum of cascade product, 2‐methoxy‐N(1‐phenoxy‐2‐propanyl)acetamide. **Supporting Fig. S26**: ^13^C NMR spectrum of cascade product, 2‐methoxy‐N(1‐phenoxy‐2‐propanyl)acetamide. **Supporting Fig. S27**: ^1^H‐^1^H COSY NMR spectrum of cascade product, 2‐methoxy‐N(1‐phenoxy‐2 propanyl)acetamide. **Supporting** **Fig.** **S28**: HPLC chromatograms for the cascade reaction. Reaction mixture after ArRmut11 step in blue and reaction mixture after CALB step in orange. Peak at 14.2 min corresponds to intermediate amine, phenoxy‐2‐propanamine. Peak at 18.5 min corresponds to ketone substrate, phenoxy‐2 propanone. Peak at 19.0 min corresponds to amide product, 2‐methoxy‐N(1‐phenoxy‐2 propanyl)acetamide. **Supporting Fig. S29**: Stacked HPLC chromatograms from the reaction monitoring of the CALB step of the cascade reaction. The chromatogram in red corresponds to the reaction mixture at the end of the ArRmut11 step while the chromatograms in black correspond to time points taken during the CALB step under reduced pressure (200 mbar), showing the consumption of the intermediate amine and the formation of the amide product. Time points (30 min to 4 h) are detailed in the *z*‐axis labels. Peak at 14.2 min corresponds to intermediate amine, phenoxy‐2‐propanamine. Peak at 18.5 min corresponds to ketone substrate, phenoxy‐2‐propanone. Peak at 19.0 min corresponds to amide product, 2‐methoxy‐N(1‐phenoxy‐2‐propanyl)acetamide. **Supporting Fig. S30**: HPLC chromatograms for the no enzyme control reactions of the cascade. A) No ArRmut11 in the transamination reaction. B) No CALB in the second step of the cascade. Peak at 14.2 min corresponds to intermediate amine, phenoxy‐2‐propanamine. Peak at 18.5 min corresponds to ketone substrate, phenoxy‐2‐propanone. In the no CALB control, the reaction was subjected to the transamination reaction with ArRmut11‐EMC7528, then methyl methoxyacetate was added but no CALB.

## Author Contributions

Lisa Kennedy designed the research, isolated the biocatalysts, carried out the biocatalytic reactions, synthesised the standards, analysed the data and wrote the manuscript. Nicholas Mulholland and Andrew Gomm designed the research and contributed to the manuscript. Dominic J. Campopiano designed the research, analysed the data and wrote the manuscript.

## Funding

This work was supported by the Engineering and Physical Sciences Research Council (EPSRC, grant EasiCAT), the University of Edinburgh and the Industrial Biotechnology innovation Centre (IBioIC).

## Conflicts of Interest

The authors declare no conflicts of interest.

## Supporting information

Supplementary Material

## Data Availability

The experimental data are included in the Supporting Information, and the primary data (NMR, HPLC, mass spectrometry) are available upon request.
