# Supplementary material for: Biocatalysis Under Reduced Pressure; Two‐Step, One‐Pot Amide Synthesis Using an Immobilised Transaminase/Lipase Cascade in Combination With By‐Product Removal
Source: Chembiochem. 2026 Mar 26;27(6):e202500951. doi: 10.1002/cbic.202500951 (PMC13022449; doi:10.1002/cbic.202500951)
Supplement: Supplementary file 1 — Supplementary Material [file CBIC-27-e202500951-s001.pdf]

Supplementary Information

Biocatalysis Under Reduced Pressure; Two-Step, One-Pot Amide Synthesis using an Immobilised Transaminase/Lipase Cascade in combination with By-Product Removal.

Lisa Kennedy, Nick Mulholland, Andrew Gomm and Dominic J. Campopiano

[a] Lisa Kennedy, Prof. Dominic Campopiano
School of Chemistry
University of Edinburgh
Joseph Black Building, David Brewster Road, Edinburgh, UK
E-mail: Dominic.Campopiano@ed.ac.uk

[b] Dr. Nicholas Mulholland, Dr. Andrew Gomm
Syngenta UK
Jealott's Hill International Research Centre, Bracknell, Berkshire, UK.

Table of Contents

Materials 4

Analytical Methods 4

NMR spectroscopy 4

Mass Spectrometry 4

High Performance Liquid Chromatography 5

Protein Purification 5

HPLC methods 5

Experimental methods 7

CALB-catalysed Kinetic Resolution (KR) 7

KR of 1-phenylethylamine by CALB under reduced pressure 7

NMR monitoring of the KR of 1-phenylethylamine by CALB lipase 8

Control reactions for the KR 9

Synthesis of 2-methoxy-N-(1-phenylethyl)-acetamide as a racemic standard for chiral HPLC analysis[5] 9

Protein expression and purification 10

Expression of ArRmut11 10

Purification of ArRmut11 10

ArRmut11 Molecular Docking 12

Substrate and amine donor screening – ArRmut11 in solution 13

General procedure for analytical scale reactions 13

Immobilisation of ArRmut11 14

CapiPy (Computer Assistance for Protein Immobilisation – Python)[7] 14

General Procedure for immobilisation of ArRmut11 15

Solvent exchange of immobilised ArRmut11 16

Assaying immobilised ArRmut11 (resin screening) 16

Marfey’s derivatisation of chiral amines 18

Synthesis of Marfey’s reagent 18

Derivatisation protocol for chiral amines 18

Cascade engineering 20

Solvent Screening 20

CALB Water Content Analysis 21

ArRmut11 Water Content Analysis 21

ArRmut11-EMC7528 Reusability Study 22

Preparative scale cascade reaction 23

Comparison of CALB step of cascade at atmospheric pressure vs 200 mbar 25

Synthesis of cascade intermediate, phenoxy-2-propanamine, standard ... 26
Chiral HPLC Chromatograms ... 28
NMR spectra ... 31
HPLC chromatograms ... 42
References ... 45

Materials

All chemicals and solvents were purchased from Sigma Aldrich or Fisher and used as received, without further purification.

Lipase B Candida antarctica immobilised on Immobead 150, recombinant from yeast (>2000 U/g) was purchased from Sigma Aldrich. 1 U corresponds to the amount of enzyme which liberates 1 µmol butyric acid per minute at pH 7.5 and 40°C (tributyrin, Cat. No. 91010, as substrate)

NPP.HCl was synthesised as described by McKenna *et al.*[1]

A Buchi Rotavapor® R-100 rotary evaporator and Buchi Vacuum Pump V-100 were used in the reactions carried out under reduced pressure.

Analytical Methods

NMR spectroscopy

^{1}H and ^{13}C NMR spectra were recorded in deuterated chloroform ($CDCl_3$), on a 400 or 500 MHz Bruker Spectrometer. The spectra have been referenced with the appropriate residual solvent peaks ($CDCl_3$ 7.26 ppm) and the coupling constants are reported to the nearest 0.1 Hz. Chemical shifts of NMR spectra are reported in parts per million (ppm) on the δ scale. Data are reported in the following way for ^{1}H NMR spectra: chemical shift, integration, multiplicity (s = singlet, d = doublet, t = triplet, q = quartet, and m = multiplet) and coupling constant in Hertz (Hz).

Mass Spectrometry

Liquid chromatography mass spectrometry (LC ESI-MS) was performed on an Agilent LC-MS TOF instrument with Zorbax C18 1.8 µm LC column coupled to an Electrospray ionisation (ESI) source. Samples were made up to 0.1 mM in acetonitrile/water (1:1) and 1 µL was injected onto the column. Elution followed a gradient 5-95% MeCN, 0.1% v/v formic acid and H_2O, 0.1% v/v formic acid over a 4.5 min period. Results were analysed with Mnova.

Protein liquid chromatography mass spectrometry (LC ESI-MS) was performed in the School of Chemistry's MS facility on a Synapt G2-Si Q-TOF (Waters) instrument with Phenomenex C4 3.6 µm LC column coupled to an ESI source. The nanodrop was used to determine protein concentration which was diluted to a final concentration of 5 µM as required. 5 µL was injected onto the column. Elution followed a gradient 5-95% MeCN, 0.1% v/v formic acid and H_2O, 0.1% v/v formic acid over a 12 min period.

High Performance Liquid Chromatography

HPLC analysis was carried out using a Shimadzu instrument fitted with an autosampler (SIL-20A HT), pump (LC-20AD), UV/visible detector (SPD-20A), system controller (CBM-20Alite) and a column oven (CTO-40C).

Protein Purification

All protein purification steps unless otherwise stated were completed on AKTA purifier Cytiva Lifesciences. Thermoscientific Heraeus Multifuge X3R was used during protein growth and purification steps with 8 × 50 mL rotor and T x 1000 mL rotor at 4 °C.

HPLC methods

HPLC analysis was carried out using one of the following methods. The method used in each experiment is listed in the experimental procedure for that experiment. Retention time is referred to as t_R.

HPLC conditions A:

**Column**	Luna 5 um C-18 column (250 x 4.6 mm)	
**Column temperature**	30 °C	
**Flow rate**	1 mL/min	
**Injection volume**	10 µL	
**Detection wavelength**	210 nm	
**Mobile phase**	A: Water (0.1% TFA)<br>B: Acetonitrile (0.1% TFA)	
**Gradient elution**	**Time (min)**	**%B Mobile Phase**
	0	5
	3	5
	6	15
	18	72
	21	72
	23	5
	27	5

HPLC conditions B:

**Column**	ChiralPak IB N-5 column (250 x 4.6 mm)	
**Column temperature**	40 °C	
**Flow rate**	1 mL/min	
**Injection volume**	10 µL	
**Detection wavelength**	254 nm	
**Mobile phase**	A: Hexane (0.1% triethylamine)<br>B: Isopropanol	
**Isocratic elution**	**Time (min)**	**%B Mobile Phase**
	15	10

HPLC conditions C:

**Column**	Luna 5 um C-18 column (250 x 4.6 mm)	
**Column temperature**	30 °C	
**Flow rate**	1 mL/min	
**Injection volume**	10 µL	
**Detection wavelength**	340 nm	
**Mobile phase**	A: Water (0.1% TFA)<br>B: Acetonitrile (0.1% TFA)	
**Isocratic elution**	**Time (min)**	**%B Mobile Phase**
	20	55

HPLC conditions D:

**Column**	ChiralPak IB N-5 column (250 x 4.6 mm)	
**Column temperature**	30 °C	
**Flow rate**	0.5 mL/min	
**Injection volume**	10 µL	
**Detection wavelength**	210, 254 nm	
**Mobile phase**	A: Water<br>B: Acetonitrile	
**Isocratic elution**	**Time (min)**	**%B Mobile Phase**
	0	35
	17	55
	20	55
	21	35
	25	35

Experimental methods

CALB-catalysed Kinetic Resolution (KR)

KR of 1-phenylethylamine by CALB under reduced pressure

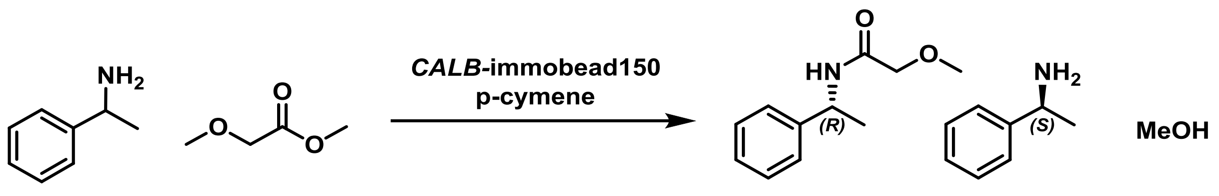

Immobilised CALB lipase (250 mg, immobead150, >2000 U/g) was added to a solution of 1-phenylethylamine (**1**) (0.9 ml, 7 mmol) and methyl methoxyacetate (**2**) (1.38 mL, 14 mmol, 2 eq.) in p-cymene (5 mL) in a round-bottom flask. The flask was placed on the rotary evaporator (rotavap) at 40 °C water bath, 200 mbar pressure, 100 rpm. At reaction completion, the flask was taken off the rotary evaporator. The reaction was stopped by filtering the immobilised CALB out of the solution. The immobilised CALB resin was washed with methanol, before drying and storing at 4 °C for reuse.

To the filtrate, an aqueous solution of HCl (1M, 5-10 drops) was added. The solution became cloudy due to the formation of (*S*)-1-phenylethylamine.HCl. This was extracted using deionised water (5 mL x 3). The organic layer was dried using $NaSO_4$, filtered, and concentrated under reduced pressure to yield a mixture of (*R*)-2-methoxy-N-(1-phenylethyl)-acetamide (**3**) and p-cymene. Due to its very high boiling point (177 °C), p-cymene is difficult to remove by rotary evaporation. The mixture of 2-methoxy-N-(1-phenylethylacetamide)/p-cymene was placed on ice and the amide product crashed out of p-cymene. The product was filtered to remove p-cymene and the crystals could be isolated in 40% yield. The combined aqueous layers from the extraction were basified using NaOH (5 M, 10 mL approx.) to pH 11. The amine product was extracted using diethyl ether (10 mL x 3). The organic layer was dried under $NaSO_4$, filtered and concentrated to yield (*S*)-1-phenylethylamine (**1**), a yellow oil (32% yield (maximum yield 50 % KR)).

(*R*)-2-Methoxy-N-(1-phenylethyl)acetamide

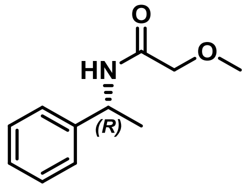

White solid (540 mg, 40% yield). 97% *ee*. **^{1}H-NMR:** (500 MHz, $CDCl_3$, ppm) δ 7.39 – 7.11 (m, 5H), 6.80 – 6.75 (br, 1H), 5.24 – 5.17 (m, 1H), 3.96 (d, *J* = 14.9 Hz, 1H), 3.90 (d, *J* = 15 Hz, 1 H), 3.43 (s, 3H), 1.51 (d, *J* = 6.9 Hz, 3H). **^{13}C-NMR:** (125 Hz, $CDCl_3$, ppm) δ 168.7 (1C), 146.0 (1C), 128.9 (2C), 127.7 (1C), 126.7 (2C), 72.1 (1C), 59.3 (1C), 48.1 (1C), 24.2 (1C). **LC-MS (ESI)** (m/z): Calculated $C_{11}H_{15}NO_2$ $[M+H]^+$: 194.1176, 195.1208; found: 194.1179, 195.1213. The enantiomeric excess was determined using HPLC method B, t_R (*R*) 10.0 min (major), t_R (*S*) 12.2 min (minor). The spectroscopic data is in accordance with literature.[2-3]

(*S*)-1-phenylethylamine

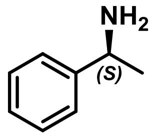

Yellow oil (271 mg, 32% yield (max. 50%)), 99% *ee*. **^{1}H-NMR** (500 MHz, $CDCl_3$, ppm) δ 7.38 – 7.33 (m, 3H), 7.29 – 7.23 (m, 2H), 4.14 (q, *J* = 6.65 Hz, 1H), 1.42 (d, *J* = 6.6 Hz, 3H). **^{13}C-NMR** (125 Hz, $CDCl_3$, ppm) δ 147.7 (1C), 128.4 (2C), 126.7 (1C), 125.6 (2C), 51.2 (1C), 25.5 (1C). The enantiomeric excess was determined by HPLC conditions B, t_R (*R*) 7.6 min (minor), t_R (*S*) 8.4 min (major). The spectroscopic data is in accordance with literature.[4]

NMR monitoring of the KR of 1-phenylethylamine by CALB lipase

The reaction was set-up as described in the general procedure, however a two-armed round-bottom flask (25 mL) was used with one arm connected to the rotavap and the other stopped with a rubber stopper. Samples (100 µL) of the reaction were taken at each time interval by briefly stopping rotation of the flask on the rotavap and inserting a needle into the rubber stopper of the second arm of the flask. In this way the pressure was maintained at 200 mbar throughout the reaction. * The samples were then diluted in d-chloroform (0.7 mL) and analysed by ^{1}H-NMR. The relative concentrations of each of the species of interest (amine and amide in the KR) were calculated using the formula below. This gave an indication of the progress of the reaction where an equal integration ratio (50:50) indicated reaction completion as 50% of the racemic amine had been consumed.

*If, after some time, the amide product of the KR precipitated out of solution, the flask was taken off the rotary evaporator and a 0.1 g sample taken using a spatula. This was then dissolved in $CDCl_3$ (0.7 mL) for NMR analysis.

Relative concentration determination

The molar ratio Mx/My between two compounds x and y was determined using the formula:

$$\frac{Mx}{My} = \frac{Ix}{Iy} \times \frac{Ny}{Nx}$$

where I is the integral, and N is the number of nuclei giving rise to the signal.

Control reactions for the KR

Control under atmospheric pressure: The KR was repeated as described and placed on the rotary evaporator; however, no vacuum was applied to the system, ie: the reaction occurred at atmospheric pressure. To monitor the reaction over time, the reaction was set-up as described in the NMR monitoring procedure above.

No enzyme control: The reaction was set-up as described in the general method but no CALB was added to the flask. The flask was placed on the rotary evaporator at 200 mbar, bath temp 40 °C, 100 rpm. The control reaction was analysed by NMR after 90 minutes and no amide formation was observed.

Synthesis of 2-methoxy-N-(1-phenylethyl)-acetamide as a racemic standard for chiral HPLC analysis[5]

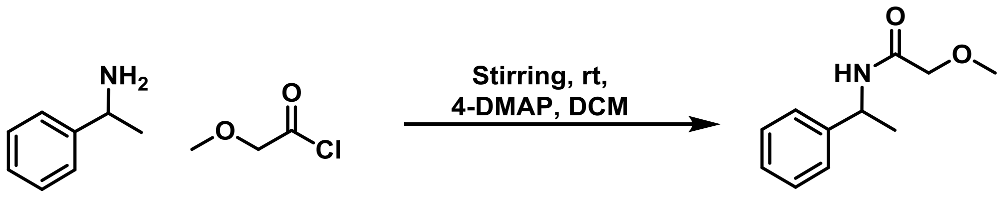

(±)-1 2 (2 eq.) (±)-3

To a stirring solution of 1-phenylethylamine (96 µL, 0.75 mmol, 1 eq.) in dichloromethane (DCM, 2 mL), methoxyacetyl chloride (0.137 mL, 1.5 mmol, 2 eq.) and 4-dimethylaminopyridine (DMAP, 0.183 g, 1.5 mmol, 2 eq.) were added in small portions. A condenser was fitted on the flask to prevent evaporation of the DCM. The reaction solution was stirred at room temperature for 4 hours. The reaction mixture was then washed with diluted HCl (1M, 5 mL x 3) and the organic layer extracted in a separatory funnel. The combined organic layers were dried over $NaSO_4$ and concentrated on the rotary evaporator. The crude white solid (87.5 mg) was recrystallised to yield a white crystalline solid (66 mg, 45% yield).

Protein expression and purification

Expression of ArRmut11

The ArRmut11 amino acid sequence (UNIPROT code: F7J696), described by Saville *et al*,[6] was used to generate a ArRmut11-encoding gene sequence optimised for *E. coli* expression. The gene was cloned in a pET28a plasmid (Genscript) containing an N-terminal 6xhistidine tag. *E.coli* BL21 (DE3) competent cells (10 µL) were transformed via heat shock transformation with plasmid DNA (2 µL) and selection was carried out on an LB agar plate containing kanamycin (30 µg/mL). The plate was incubated overnight at 37 °C. One colony was used to inoculate a starter culture LB media (250 mL) containing kanamycin (30 µg/mL) and incubated overnight at 37 °C, 200 rpm. The starter culture inoculated LB media (1 L) containing kanamycin (30 µg/mL) to an OD600 of 0.1. The cells were grown at 37 °C, 200 rpm until an OD600 of 0.6 - 1.0 was achieved. Protein expression was induced using iso-propyl-β-D-1-thiogalactopyranoside (IPTG) (final concentration 0.2 mM). The temperature was lowered to 16 °C, 180 rpm overnight. The cells were harvested by centrifugation (Thermo Scientific Multicentrifuge X3R, 3500 x g, 20 min, 4 °C, 4 x 1000 rotor). The cell pellets were resuspended in phosphate buffer, centrifuged (Thermo Scientific Multicentrifuge X3R, 4000 x g, 45 min, 4 °C, 8 x 50 rotor) and cell pellets were stored at -20 °C.

Purification of ArRmut11

The cell pellet was defrosted on ice and resuspended in binding buffer (sodium phosphate (pH 7.4, 50 mM), NaCl (300 mM), imidazole (20 mM), PLP (0.1 mM)). Cell lysis was carried out by sonication (30 s on 30 s off, 15 cycles) and the cell debris was collected by centrifugation (Thermo Scientific Multicentrifuge X3R, 9000 x g, 50 min, 4 °C, 8 x 50 rotor). The cell lysate was filtered using Millex HA filters (0.45 µm) and loaded onto a pre-equilibrated His Trap Nickel affinity column (5 mL) using an ÄKTA explorer (Cytiva Lifesciences, UK) monitoring at 280 nm. The column was washed with 20 column volumes of binding buffer (sodium phosphate (pH 7.4, 50 mM), NaCl (300 mM), imidazole (20 mM), PLP (0.1 mM)). Elution buffer (sodium phosphate (pH 7.4, 50 mM), NaCl (300 mM), imidazole (300 mM), PLP (0.1 mM)) was applied with a gradient of 0 to 100% over 10 min then held at 100% for 10 min, 5 mL/min. Analysis of fractions with high UV/Vis absorbance by 12% SDS-PAGE was carried out. Collected fractions were concentrated and dialysed overnight in dialysis tubing (8000 MWCO) in dialysis buffer (sodium phosphate (pH 7.4, 50 mM), NaCl (300 mM), PLP (0.1 mM)). Protein concentration was determined by nanodrop (extinction coefficient = 51,005 M^{-1} cm^{-1})

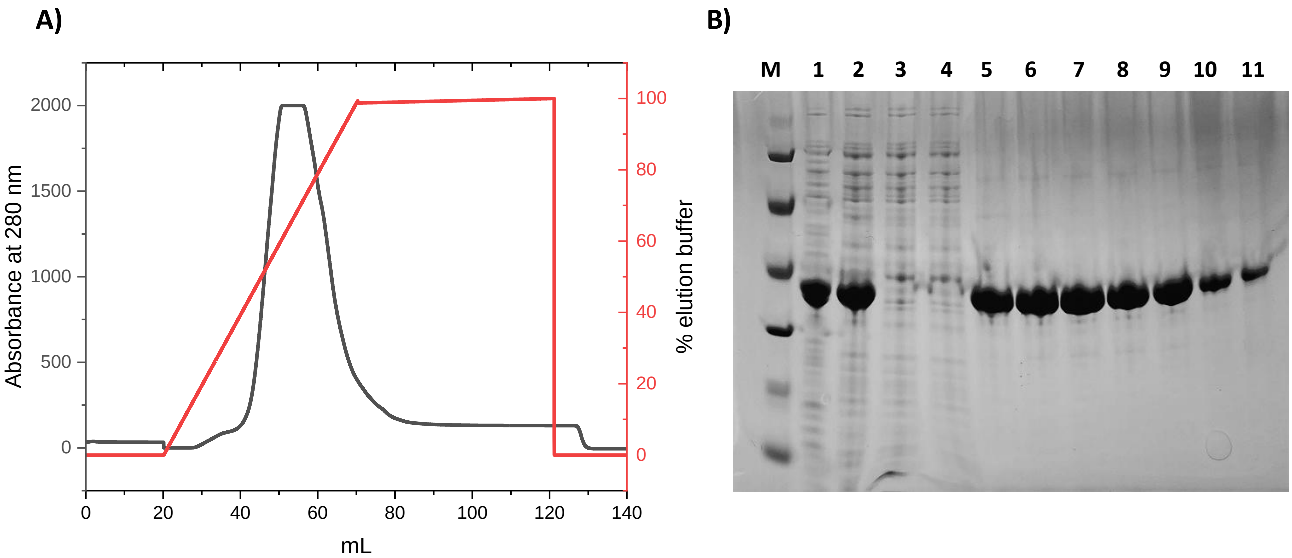

Figure S1. A) Elution profile of the immobilised metal affinity chromatography (IMAC) during purification of ArRmut11. B) SDS gel analysis of purified fractions; M = marker, 1 = cell pellet, 2 = lysate, 3 = flow through during loading, 4 = flow through during column wash, 5-11 = IMAC fractions. Expression conditions: LB media (1L cultures), kanamycin 30 ug/ml, IPTG 0.2 mM, 20 °C overnight. Yield = 74 mg protein/1L culture.

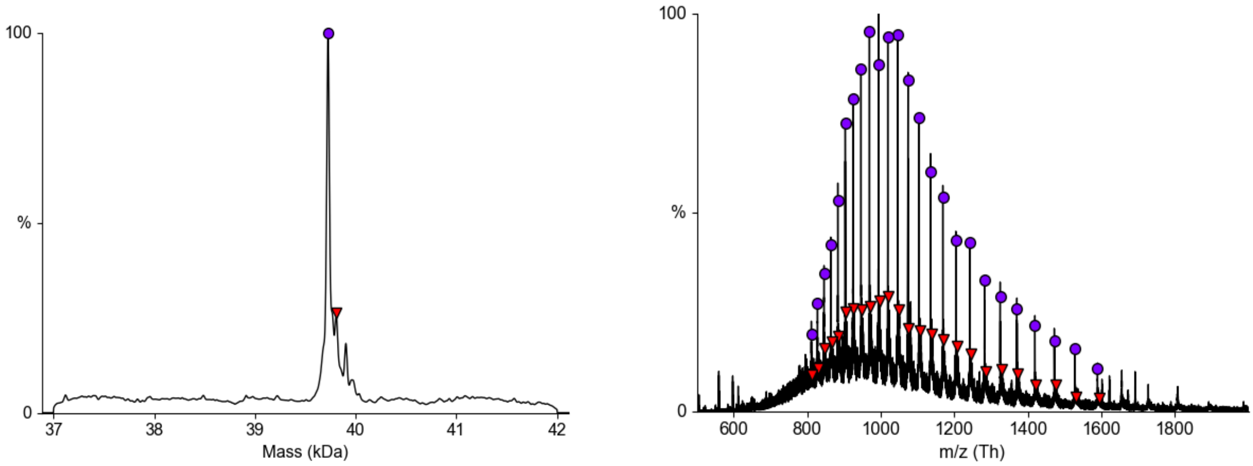

Figure S2. ESI-MS spectrum of purified ArRmut11. Calculated mass = 39726.24 ± 0.58 Da which corresponds to the predicted mass minus the N-terminal methionine residue (39725.47 Da).

ArRmut11 Molecular Docking

The crystal structure of ArRmut11 published in the PDB (5FR9) was used for docking experiments. The ArRmut11 binding site was identified by topological analysis via the CASTp 3.0 server, using a probe radius of 1.4 Å. The ligands (acetophenone, 4-phenyl-2-butanone and phenoxy-2-propanone) and ArRmut11 receptor were prepared using AutoDockTools (v.1.5.7), and ligand docking was performed using AutoDock Vina (v1.1.2).[11-12] The docking experiment was repeated up to 10 times with gradually increasing exhaustiveness (8-128). The top-ranked pose from each docking experiment was sampled for visualisation and analysis in PyMOL (v2.4.0).

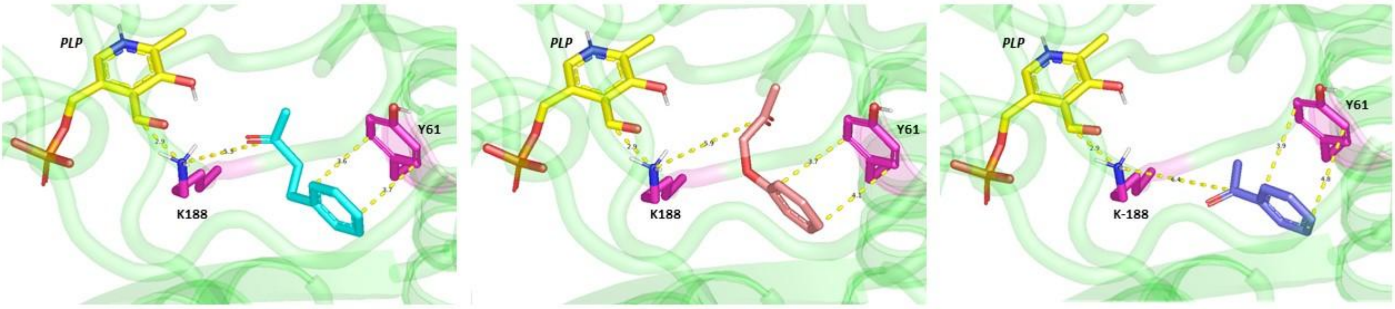

Figure S3. 4-phenyl-2-butanone (cyan), phenoxy-2-propanone, 3 (peach) and acetophenone, 1 (indigo) docked in the active site of ArRmut11 (PDB code = 5FR9). PLP shown in yellow, residues Lys-188 and Tyr-61 shown in pink. The pi-pi stacking interaction seen with the tyrosine may be promoting better binding for the 4-carbon chain substrates than that seen with acetophenone. This interaction holds the ketone molecule in the optimal area for reacting.

Substrate and amine donor screening – ArRmut11 in solution

General procedure for analytical scale reactions

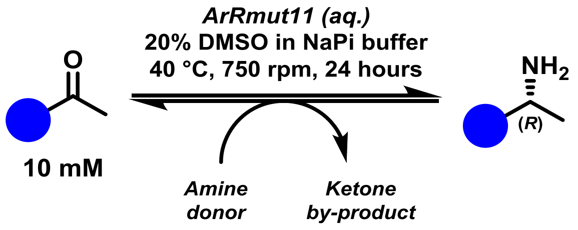

Ketone stocks were prepared at a concentration of 100 mM in DMSO. Isopropylamine and NPP.HCl stocks were prepared in buffer at a concentration of 5 M or 200 mM respectively. Reactions were set up at 10 mM ketone concentration at 0.5 mL scale with 20% DMSO (Ketones screened shown in Table 1). Ketone (50 µL, 100 mM), amine donor (50 µL) and ArRmut11 (74 µL, 6.8 mg/mL) were added to a clean Eppendorf tube. DMSO (50 µL) and sodium phosphate buffer (50 mM, pH 8) was added to make up to 500 µL volume. The reactions took place at 40 °C, 750 rpm for 24 hours. After this, the reactions were centrifuged (13000 xg, 10 mins) and 100 µL supernatant was diluted in 900 µL HPLC grade water for analysis by HPLC using method A.

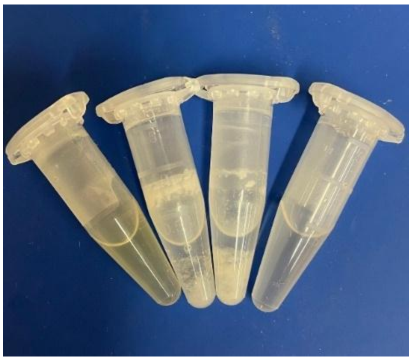

Figure S4. Example of NPP dimer precipitating in reactions when using NPP as amine donor for ArRmut11.

Immobilisation of ArRmut11

CapiPy (Computer Assistance for Protein Immobilisation – Python)[7]

The CapiPy package was run in an anaconda environment following the instructions in the GitHub file.[7] The PDB code (5FR9) for ArRmut11 was used to retrieve the crystal structure and sequence. CapiPy performed a surface residue analysis on the dimeric structure and clustered residues into one of 4 groups: hydrophobic, positively charged, negatively charged and histidine clusters.

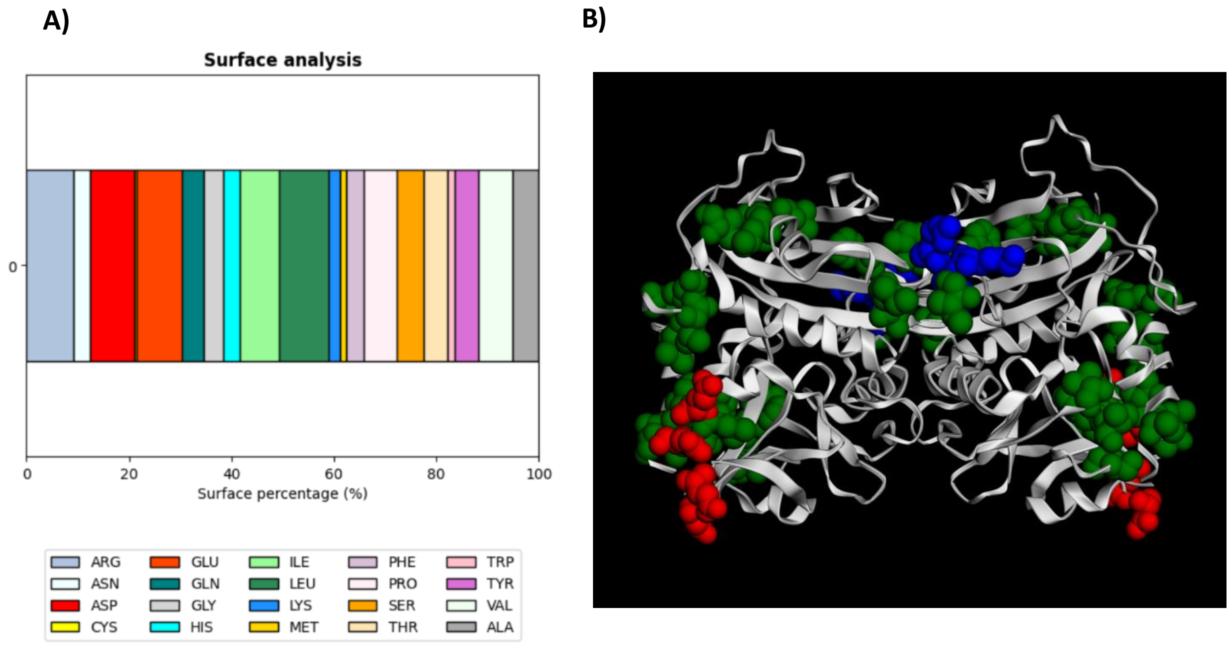

Figure S5. CapiPy results for ArRmut11. A). Surface residue analysis showing abundance of each amino acid residue on the exposed surface of the protein. B). Physical representation of surface exposed residues on the structure of ArRmut11 (PDB code: 5FR9), where clusters of amino acids are coloured green = hydrophobic, red = negative, blue = positive.

General Procedure for immobilisation of ArRmut11

The protocol was adapted from the SunResin protocol for immobilisation on epoxy or absorbent resins (SunResin; EMC7528, EMC7014. Purolite; ECR8285, ECR8204, ECR8806). As a standard measurement, the protein loading was equivalent to 20 mg protein per 1 g of resin.

The metal affinity resin (Chelex7350) should be pre-loaded with Ni prior to immobilisation. This can be done by mixing the resin with a solution of $NiCl_2$ (0.35 mmol per mL, 1:4 w/v resin/solution) for 3 hours, then filtering and washing with water prior to carrying out the immobilisation protocol.

1. **Resin Equilibration:**

The resin was equilibrated by washing with immobilisation buffer (sodium phosphate 50 mM, pH 8) in a ratio of 1:4 (w/v) resin/buffer. The resins were filtered and the filtrate was discarded. This was repeated 2 – 3 times and the wet resins were collected and stored at 4 °C until immobilisation.

2. **Preparation of the enzyme solution:**

A solution of ArRmut11 was prepared to have a protein loading of 20 mg per g resin in the immobilisation step. As ArRmut11 is a PLP-dependent transaminase, the solution also contained PLP (0.1 mM). The native enzymes were dissolved in immobilisation buffer to achieve a ratio of 1:4 (w/v) resin/solution in the immobilisation step. The protein concentration of the enzyme solution was confirmed using the nanodrop prior to immobilisation.

3. **Immobilisation:**

The enzyme solution was transferred to falcon tubes along with the equilibrated resin achieving a ratio of 1:4 (w/v) resin/enzyme solution. The resin/enzyme solution was mixed gently (to avoid air bubbles) for 24 hours at room temperature. For the epoxy resins, the mixtures were then left stationary at room temperature for a further 24 hours.

4. **Filtration and Washing**

The resin/enzyme solution was filtered under vacuum. The filtrates were collected and the protein concentration was measured using Nanodrop to calculate the immobilisation yield. The filtered immobilised enzymes were washed with ice-cold water and dried under vacuum. The immobilised enzymes were then stored at 4 °C until use.

Solvent exchange of immobilised ArRmut11

The immobilised ArRmut11 was washed with isopropanol/PEG-400/water (85:10:5) (2:1 solution/resin).[8] This was repeated twice, the resin was filtered and allowed dry under vacuum in the fumehood for up to 1 hour before use in organic media.

Assaying immobilised ArRmut11 (resin screening)

To screen the various resins for activity, each was tested on an analytical scale in both aqueous and organic media.

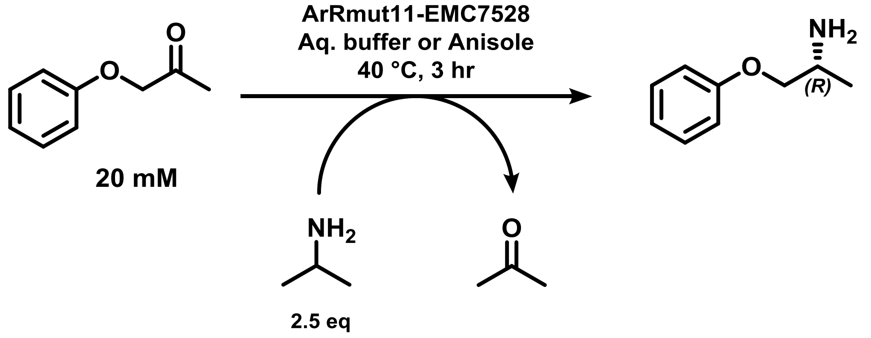

Aqueous media reactions:

ArRmut11 immobilised on resin (50 mg, enzyme loading = 20 mg/g resin) was weighed out and placed in an Eppendorf tube. Sodium phosphate buffer (700 µL, 50 mM, pH 8) was added. To this, phenoxy-2-propanone (200 µL, 100 mM stock in DMSO) and isopropylamine (100 µL, 500 mM stock in H_2O) were added. Reactions were set up in triplicate for each resin. Reactions were left at 45 °C, 800 rpm for 3 hours. After this time, a sample (50 µL) of the reaction solution was taken and diluted in 950 µL HPLC water for HPLC analysis using method A and quantified through calibration curve or the ketone substrate. The error was calculated as standard deviation.

Organic media reactions:

Each resin was weighed out (150 mg) and solvent exchanged by washing with a mixture of isopropanol/PEG-400/H_2O (85:10:5) and allowed to dry in the fumehood for 1 hour before use. The solvent exchanged resin was divided between 3 test tubes for the triplicate reactions (3 x 50 mg). Anisole (700 µL) was added to each tube. To this, phenoxy-2-propanone (200 µL, 100 mM stock in anisole) and isopropylamine (100 µL, 500 mM stock in anisole) were added. The reactions were left at 45 °C, 800 rpm for 3 hours. After this time, a sample (50 µL) of the reaction solution was taken and diluted in 950 µL HPLC ACN for HPLC analysis using method A and quantified through calibration curve or the ketone substrate. The error was calculated as standard deviation.

Phenoxy-2-propanone

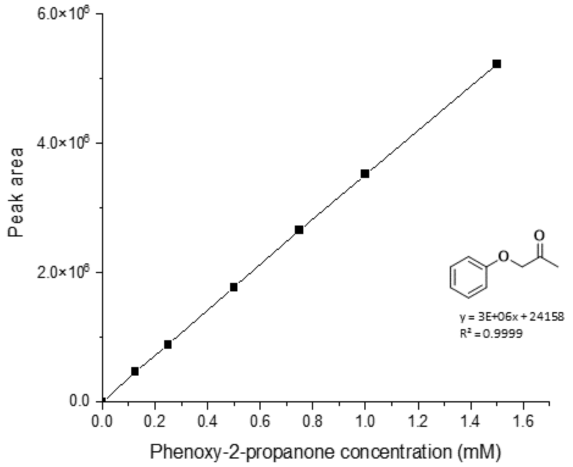

Figure S6. Phenoxy-2-propanone calibration curve produced by HPLC analysis of phenoxy-2-propanone at concentrations 0 – 1.5 mM using HPLC method A (t_R = 19.5 min). Samples of each concentration were run in triplicates and the average peak area plotted. The error was determined as the standard deviation.

Phenoxy-2-propamine

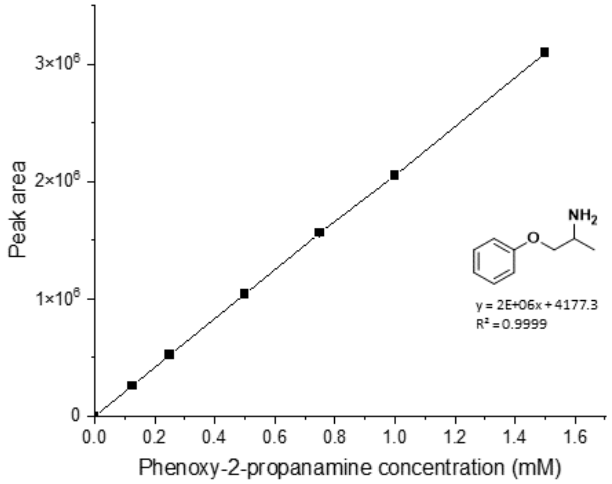

Figure S7. Phenoxy-2-propamine calibration curve produced by HPLC analysis of phenoxy-2-propamine at concentrations 0 – 1.5 mM using HPLC method A (t_R = 14.2 min). Samples of each concentration were run in triplicates and the average peak area plotted. The error was determined as the standard deviation. The standard of phenoxy-2-propanamine used to make up a standard solution was synthesised by the procedure detailed below.

Marfey's derivatisation of chiral amines

To determine the enantiomeric excess of a chiral amine, we derivatised the amine using Marfey's reagent to form diastereomers that can be separated on a C18 HPLC column.

Synthesis of Marfey's reagent

Marfey's reagent was prepared following the procedure reported by Lanigan *et. al.*, in 2016.[9]

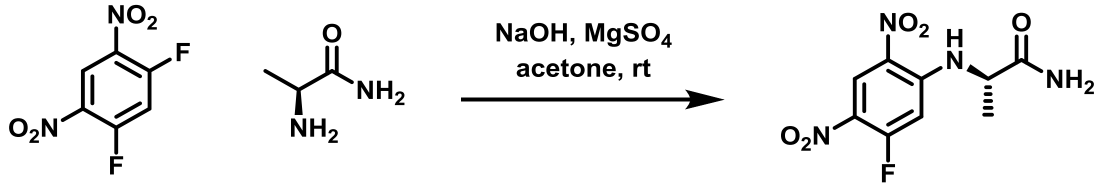

L-alaninamide (0.236 g, 2.6 mmol) was dissolved in 1M NaOH (1.85 mL) with stirring. Acetone (30 mL) was added and the mixture was stirred for 5 minutes at room temperature. $MgSO_4$ (5 g) was added. The reaction mixture was stirred for 3 hours at rt. After 3 hours, the $MgSO_4$ was filtered off. 1,5-difluoro-2,4-dinitrobenzene (0.334 g, 1.6 mmol) was dissolved in acetone (7.5 mL) with stirring. The L-alaninamide solution was added to this dropwise. It was then stirred for 45 minutes. Water (40 mL) was added dropwise resulting in the formation of yellow crystals. The flask was placed in an ice bath during this process to promote crystal formation. The crystals were then filtered under vacuum and washed with ice cold water/acetone mixture (7.5 mL x 3) to give Marfey's reagent.

Yellow crystals (0.188 g, 41% yield). **^{1}H NMR** (500 MHz, MeOD, ppm) δ 9.10 (d, *J* = 5.2 Hz, 1H), 8.91 (d, *J* = 8.3 Hz, 1H), 7.73 (s, 1H), 7.49 (s, 1H), 6.96 (d, *J* = 14.5 Hz, 1H), 4.40 (p, *J* = 6.8 Hz, 1H), 1.46 (d, *J* = 6.8 Hz, 3H). **^{13}C NMR** (126 MHz, MeOD, ppm) δ 175.7, 162.0, 159.9, 149.2, 129.1, 128.4, 102.6, 53.4, 19.0. In accordance with literature. [9]

Derivatisation protocol for chiral amines

Amine standards (55 µL, 20 mM) was added to an Eppendorf tube. Marfey's reagent (82.5 µL, 40 mM in acetone) was added. $NaHCO_3$ (20 µL, 1 M) and water (62.5 µL) were added to make a final volume of 220 µL. The reaction was left at 40°C, 750 rpm for 1 hour. The reaction was quenched by addition of HCl (20 µL, 2 M). For HPLC analysis, 50 µL of the reaction was diluted in 250 µL ACN/Water 50:50. Analysed using HPLC method C.

Reaction in anisole (22 µL, 50 mM substrate concentration) was added to an Eppendorf tube. Marfey's reagent (165 µL, 40 mM in acetone) was added. $NaHCO_3$ (20 µL, 1 M) and acetone (13 µL) was added to make a final volume of 220 µL. The reaction was left at 40°C, 750 rpm for 1 hour. The reaction was quenched by addition of HCl (20 µL, 2 M). For HPLC analysis, 50 µL of the reaction was diluted in 250 µL ACN/Water 50:50. Analysed using HPLC method C.

A)

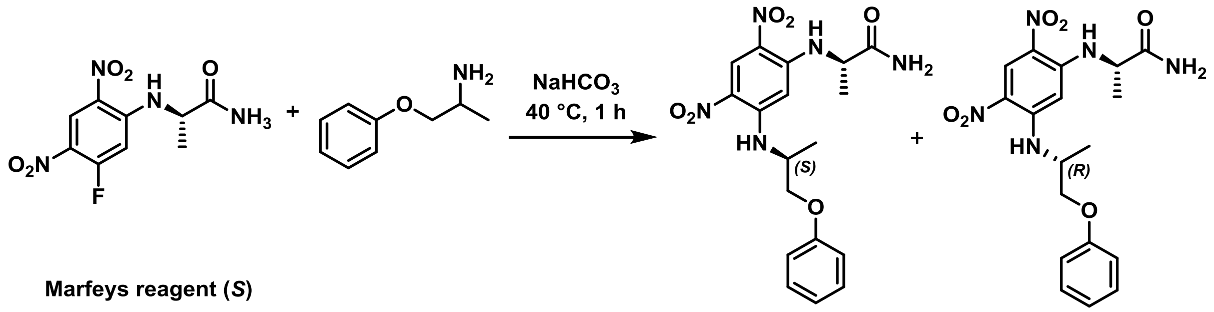

Marfeys reagent (*S*)

(*S*), (*S*)

(*S*), (*R*)

B)

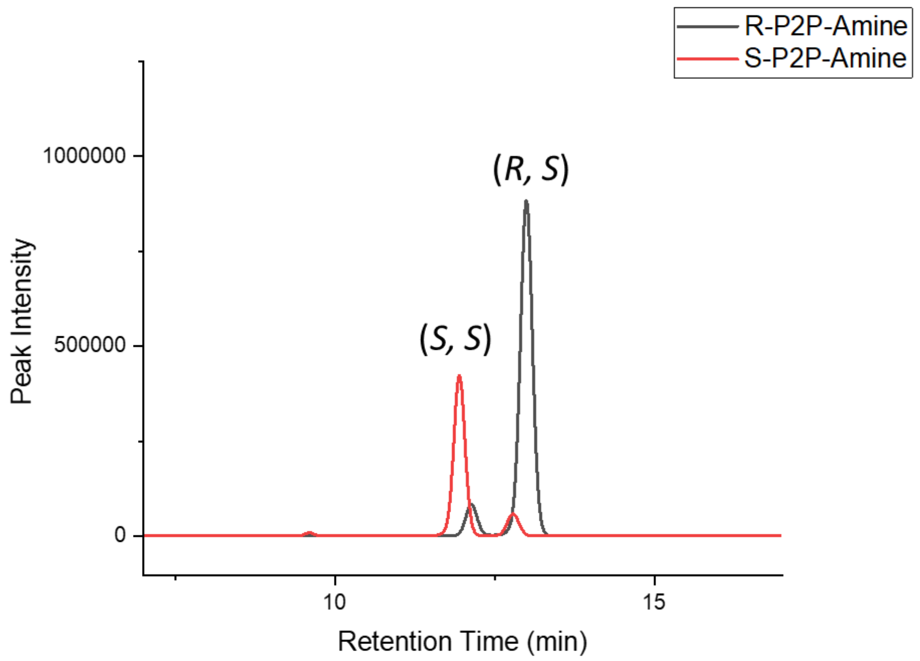

Figure S8. A) Reaction scheme for the derivitisation of phenoxy-2-propanone using Marfey's reagent. **B)** HPLC chromatograms for phenoxy-2-propanamine (P2P-Amine) derivatised with Marfey's reagent as a method for resolving enantiomers. The black line represents the chromatogram for the reaction with ArRmut11, an *R*-selective transaminase. The red line represents the chromatogram for the reaction with CVTA, an *S*-selective transaminase, used as a standard. The (*R, S*)-diastereomer produced in the derivatisation procedure elutes with t_R = 12.7 min while the (*S, S*)-diastereomer elutes with t_R = 12.0 min.

Cascade engineering

Solvent Screening

Solvents tested:

1. *p*-Cymene
2. Anisole
3. Cyrene
4. CPME
5. Dioxane
6. *tert*-Butanol
7. Methyl methoxyacteate (acyl donor as solvent)
8. Sodium phosphate buffer (50 mM, pH 7.5)

Substrate stocks for both the CALB and ATA reactions were prepared in each solvent 1 – 7 to a concentration of 100 mM (and 500 mM for ATA amine donor, isopropylamine). For reactions in buffer, stocks were prepared in DMSO. Individual ATA or lipase small-scale reactions (1 mL) were prepared in each solvent at a 10 mM substrate concentration with 3 eq. acyl donor (CALB reaction) or 2.5 eq. amine donor (ArRmut11 ATA reaction).

CALB reactions; CALB-immobead150 (10 mg, >2000 U/g) was added to each reaction and left at 40 °C, 750 rpm for 6 hrs. To analyse: 50 µL of each reaction was diluted with either 950 µL HPLC grade acetonitrile (ACN) or HPLC grade H2O depending on water miscibility of the solvent. Samples were analysed using HPLC method A. Results were quantified by a calibration curve of the product amide.

ATA reactions; A small amount of water was added to the ATA reactions to ensure water-saturation of the solvent (1% v/v). ArRmut11-EMC7528 was solvent exchanged prior to use. ArRmut11-EMC7528 (80 mg, protein loading: 15 mg/g resin) was added to each reaction. The reactions were left at 40 °C, 500 rpm for 22 hours. To analyse: 50 µL of each reaction was diluted with either 950 µL HPLC grade acetonitrile (ACN) or HPLC grade H2O depending on water miscibility of the solvent. Samples were analysed using HPLC method A. Results were quantified by a calibration curve of the product amide.

CALB Water Content Analysis

Reactions were prepared in triplicates at 1 mL final volume. (*R*)-methylbenzylamine (100 µL, 100 mM stock in anisole) and methyl methoxyacetate (300 µL, 100 mM stock in anisole) were added to an Eppendorf tube. Anisole (700 – 650 µL) and water (0 – 50 µL) were added to each of the reactions to give an added water content of 0 – 5% v/v. CALB-immobead150 (10 mg, >2000 U/g) was added to each reaction. The reactions were allowed to react for 19.5 hrs at 40 °C, 750 rpm. To analyse: 50 µL of each reaction was diluted with 950 µL HPLC grade ACN. Samples were analysed using HPLC method A. Results were quantified by a calibration curve of the product amide.

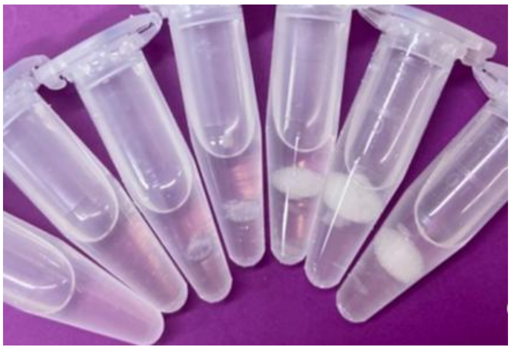

Figure S9. Water content analysis for CALB on analytical scale from 0 – 3% added water in anisole. Reactions are shown from 0% (left hand side) to 3% (right hand side). The immobilised CALB can be seen to be clumping together as the % water increases and a water bubble is formed in the solvent.

ArRmut11 Water Content Analysis

Reactions were prepared in duplicates at 1 mL final volume. Phenoxy-2-propanone (100 µL, 100 mM stock in anisole) and isopropylamine (100 µL, 5 M stock in anisole) were added to an Eppendorf tube. Anisole (800 – 750 µL) and water (0 – 50 µL) were added to each of the reactions to give an added water content of 0 – 5% and final volume of 1 mL. ArRmut11-EMC7528 was solvent exchanged before use by washing with IPA/PEG-400/Water (85:10:5) and dried in the fumehood for at least an hour. ArRmut11-EMC7528 (80 mg, protein loading: 15 mg/g resin) was added to each reaction. The reactions were allowed to react for 18 hrs at 45 °C, 750 rpm. To analyse: 50 µL of each reaction was diluted with 950 µL HPLC grade ACN. Samples were analysed using HPLC method A.

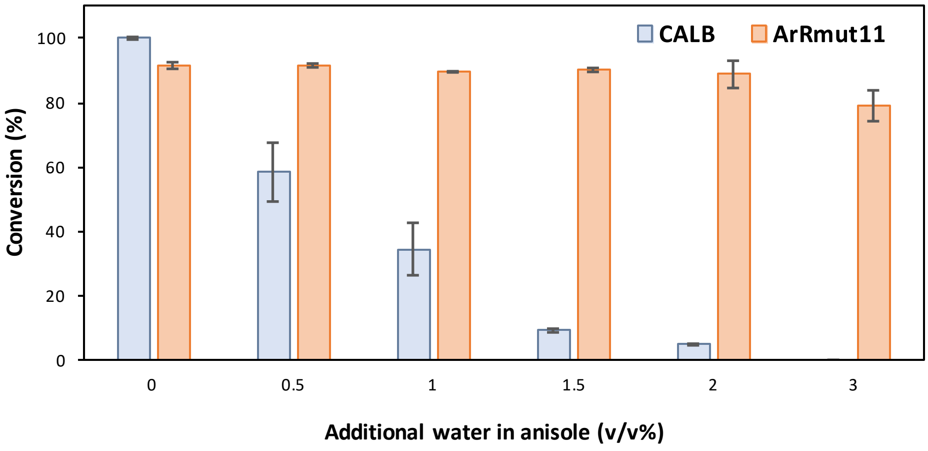

Figure S10. Effect of added water (v/v%) on the reaction in anisole for ArRmut11-EMC7528 and CALB-immobead150.

ArRmut11-EMC7528 Reusability Study

The reusability of ArRmut11-EMC7528 in anisole was studied using the transamination of phenoxy-2-propanone. Reactions were prepared in triplicates at 1 mL final volume. Phenoxy-2-propanone (100 µL, 100 mM stock in anisole) and isopropylamine (100 µL, 5 M stock in anisole) were added to an Eppendorf tube. Anisole (785 µL) and water (15 µL, 1.5% v/v) were added. ArRmut11-EMC7528 was solvent exchanged before use by washing with IPA/PEG-400/Water (85:10:5) and dried. ArRmut11-EMC7528 (80 mg, protein loading: 15 mg/g resin) was added to each reaction. The reactions were allowed to react for 24 hrs at 45 °C, 750 rpm. A 50 µL sample was taken from each reaction and diluted with 950 µL HPLC grade ACN. Samples were analysed using HPLC method A. The rest of the reaction mixture was discarded and a new reaction set-up as before with the same immobilised enzymes. This was repeated for 8 consecutive days.

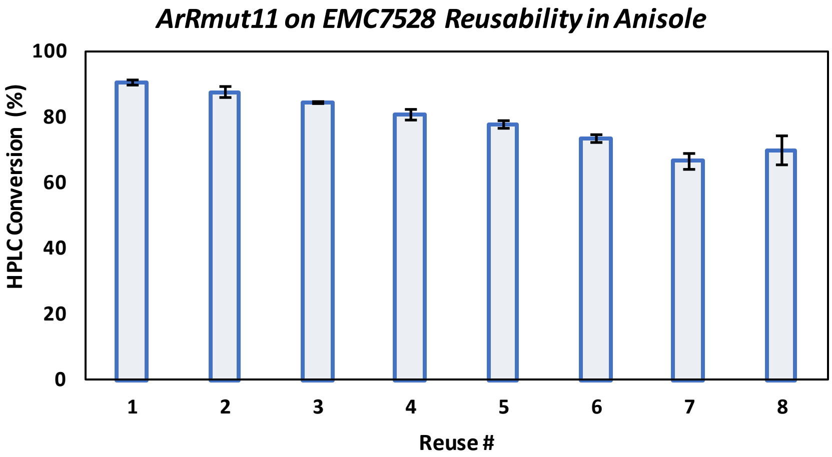

Figure S11. Bar chart showing the reaction conversion for the transamination of phenoxy-2-propanone (10 mM) by ArRmut11-EMC7528 in anisole (1.5% H_2O) over 8 consecutive uses of the same batch of immobilised enzyme. Reaction conditions detailed above. Error calculated as standard deviation of triplicate reactions.

Preparative scale cascade reaction

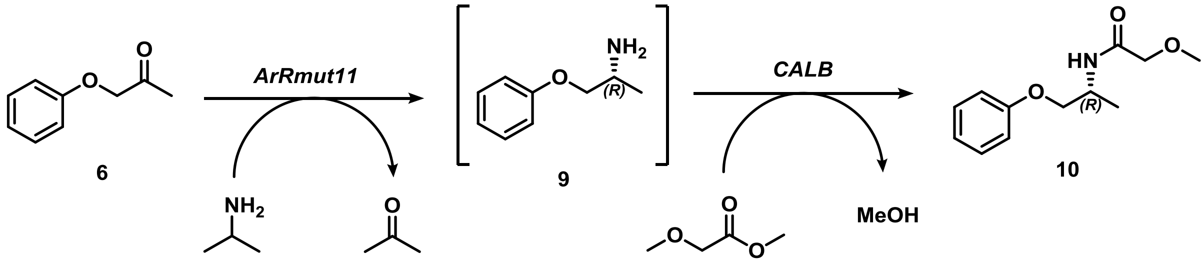

Anisole (20 mL) was added to a 50 mL round-bottom flask. To this, phenoxy-2-propanone, **6** (0.137 mL, 1 mmol) and isopropylamine (0.430 mL, 5 mmol, 5 eq.) were added. The reaction was heated to 45 °C on a hotplate and a small stir bar added. ArRmut11-EMC7528 (1 g, enzyme loading = 20 mg/g resin), which had been solvent exchanged prior to use, was added to the flask. The reaction was stirred at low rpm (approx 100 rpm) to avoid damage to the immobilised enzyme at higher speeds. The flask was stoppered to prevent evaporation of isopropylamine. The reaction was left overnight (18 hours) under these conditions. ArRmut11-EMC7528 was then filtered out of solution under vacuum. The filtrate was collected placed in a 100 mL round-bottom flask. To this, methyl methoxyacetate (0.7 mL, 7 mmol) and CALB-immobead150 (200 mg, >2000 U/g) were added. The flask was immediately placed on the rotary evaporator at 200 mbar, 40 °C water bath, 100 rpm. This was left for 4 hours before removing from the rotary evaporator and filtered off the CALB-

immobead150. The reaction was monitored by HPLC; samples were prepared by taking a sample (20 µL) and dissolving in HPLC grade acetonitrile (980 µL). Samples were analysed by HPLC method A.

Purification of cascade product

The crude reaction mixture in anisole was left on the rotary evaporator at 20 mbar for 1 hour to concentrate before purification and remove excess acyl and amine donor. The remaining mixture was purified by column chromatography hexane/ethyl acetate gradient elution 100% hexane to 100% EtOAc. The fractions containing the product were pooled and concentrated under reduced pressure to yield a yellow oil (50% yield). The product was analysed by ^{1}H- and ^{13}C-NMR and ESI-MS.

2-Methoxy-N-(1-phenoxy-2-propanyl)acetamide

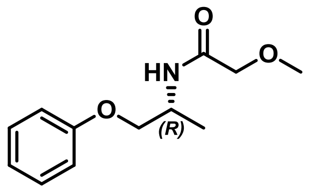

Yellow oil (50% yield, >99%*ee*). **^{1}H NMR** (500 MHz, $CDCl_3$, ppm) δ 7.32 – 7.24 (m, 2H), 6.96 (tt, *J* = 7.3, 1.1 Hz, 1H), 6.91 (dd, *J* = 8.8, 1.0 Hz, 2H), 6.75 (s, 1H), 4.43 (m, 1H), 3.97 (dd, *J* = 4.2, 1.2 Hz, 2H), 3.93 – 3.84 (m, 2H), 3.40 (s, 3H), 1.34 (d, *J* = 6.8 Hz, 3H). **^{13}C NMR** (126 MHz, $CDCl_3$, ppm) δ 169.06, 158.70, 129.53, 121.10, 114.57, 72.00, 70.45, 59.13, 44.08, 17.55. **LC-MS (ESI)** (m/z): calculated $[M+H]^+$: 224.1281, found $[M+H]^+$: 224.1276. The enantiopurity of the product was analysed using HPLC method D; t_R (*R*)-amide = 15.1 min (major), t_R (*S*)-amide = 15.5 min (minor).

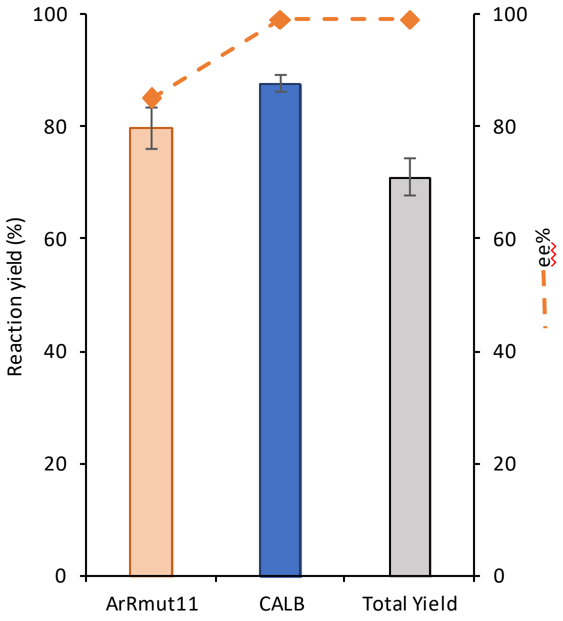

Figure S12. Yield data for the biocatalytic cascade starting from phenoxy-2-propanone (**6**) to 2-methoxy-N(1-phenoxy-2-propanyl)acetamide (**10**). Reaction yield for the transamination of **6** to **9** by

ArRmut11-EMC7528 is shown in peach. Reaction yield for the acylation of **9** to form **10** by CALB-immobead150 is shown in blue. The overall yield for the two-step cascade is shown in grey. The ee% for each reaction is shown in the orange dotted line.

Comparison of CALB step of cascade at atmospheric pressure vs 200 mbar

The first step of the cascade was set up at 20 mL scale as described above. After 18 hours, ArRmut11-EMC7528 was filtered out and the reaction mixture was split evenly in half (2 x 10 mL) and placed in two separate 50 mL two-armed round-bottom flasks. One arm of the flasks was stoppered with a rubber septum. To each 10 mL reaction, methyl methoxyacetate (3.5 mmol) and CALB-immobead150 (100 mg, >2000 U/g) were added. One flask was placed on the rotary evaporator at 200 mbar while the other was placed on a second rotary evaporator (both Buchi branded) without the vacuum switched on (atmospheric pressure). The water bath for both was set at 40 °C and the rotation to 100 rpm. To take time points, a needle was inserted through the rubber septum on the second arm of the flask and 50 µL of the reaction was withdrawn. Therefore, the pressure was kept constant throughout the reaction. From this, HPLC samples were prepared by dissolving each sample (20 µL) in HPLC grade acetonitrile (980 µL). Samples were analysed using HPLC method A.

1)

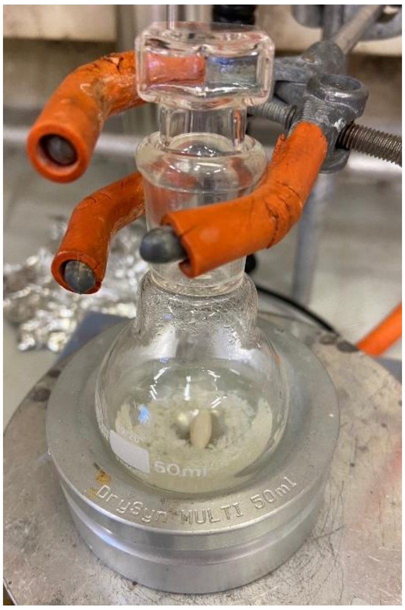

2)

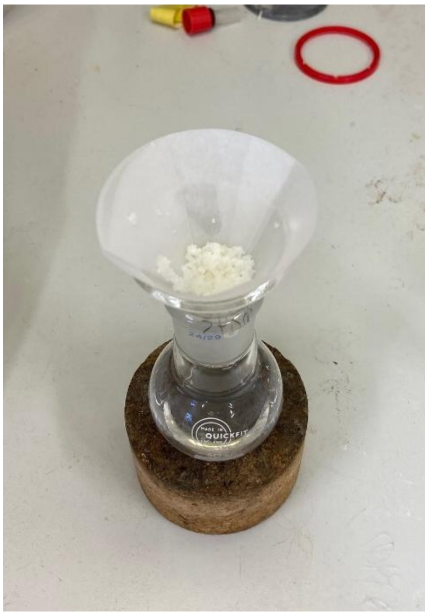

3)

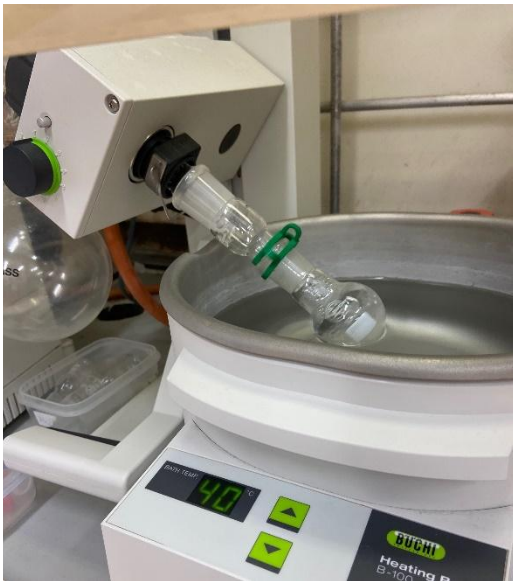

Figure S13. Step by step of cascade reaction. 1) ArRmut11 step at atmospheric pressure 2) filtering immobilised ArRmut11 out of reaction 3) CALB step on rotary evaporator at 200 mbar.

Synthesis of cascade intermediate, phenoxy-2-propanamine, standard

A standard of phenoxy-2-propanamine was synthesised using ArRmut11-EMC7528 in a scaled-up 200 mL reaction using the SpinChem rotating bed reactor. ArRmut11 was immobilised on EMC7528 as previously described. Phenoxy-2-propanone (0.55 mL, 20 mM final concentration) was dissolved in DMSO (30 mL). To this, sodium phosphate buffer (165 mL, 50 mM, pH 8), isopropylamine (3.43

mL, 10 eq.) and PLP (800 µL, 25 mM) were added. The reaction solution was transferred to the SpinChem 200mL reactor which was heated to 40 °C through a heating jacket. ArRmut11-EMC7528 (5 g, enzyme loading = 20 mg/g resin) was packed into the rotating bed reactor (RBR). The RBR was placed in the reactor and set to 200 rpm. The reaction was carried out under these conditions for 4.5 hours. After this time, the reaction solution was drained from the reactor and basified to pH 11. The amine product was extracted into ethyl acetate. The EtOAc layer was washed with 1M NaOH several times to remove DMSO, before being concentrated under reduced pressure. The concentrated solution was loaded onto a prepHPLC column in 3 batches to purify (10 – 60% acetonitrile (ACN) (0.1% TFA) over 12 min, 100% ACN (0.1% TFA) hold 5 min). The fractions containing product were freeze dried to yield an orange solid (TFA salt, 0.367 g, 61% yield, 85%*ee*). **^{1}H NMR** (500 MHz, $CDCl_3$) δ 7.31 – 7.21 (m, 2H + $CDCl_3$), 7.01 – 6.94 (m, 1H), 6.86 (d, *J* = 8.7 Hz, 2H), 4.01 (dd, *J* = 10.0, 3.7 Hz, 1H), 3.92 (dd, *J* = 10.0, 7.2 Hz, 1H), 3.60 – 3.50 (m, 1H), 1.32 (d, *J* = 6.8 Hz, 3H). **^{13}C NMR** (126 MHz, $CDCl_3$) δ 157.96 (TFA), 130.02, 122.23, 114.95, 68.64, 47.91, 15.24. **LC-MS (ESI):** calculated $[M+H]^+$: 152.1070, found $[M+H]^+$: 152.1073. In line with literature.[10]

Chiral HPLC Chromatograms

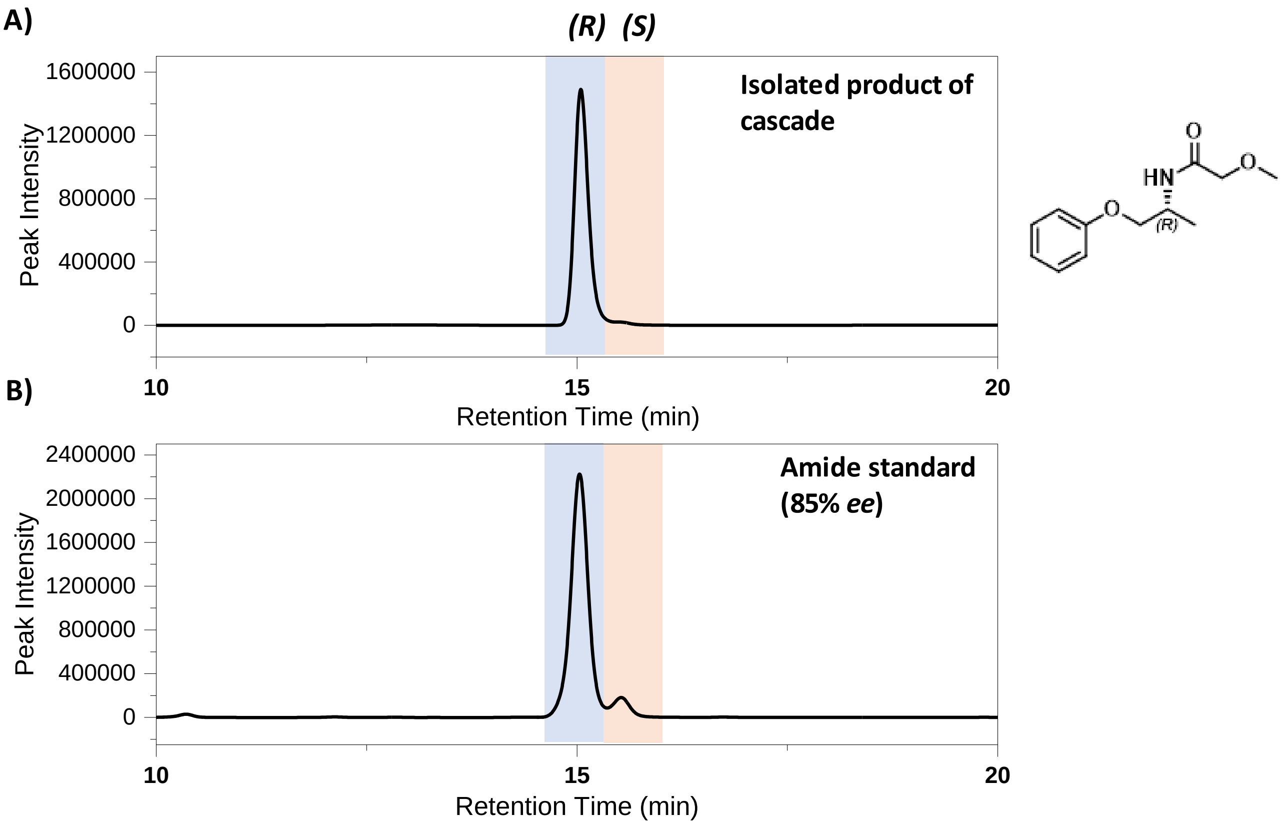

Figure S14. Chiral HPLC chromatograms for **A)** the isolated amide product, 2-Methoxy-N-(1-phenoxy-2-propanyl)acetamide, of the biocatalytic cascade, synthesised by ArRmut11 and CALB (99% *ee*). **B)** the amide product derived from chemical acylation of phenoxy-2-propanamine from the ArRmut11 reaction (85% *ee*). t_R (*R*)-amide = 15.1 min (blue), t_R (*S*)-amide = 15.5 min (peach), HPLC method D. The coupling of the two biocatalysts causes an amplification of enantiopurity.

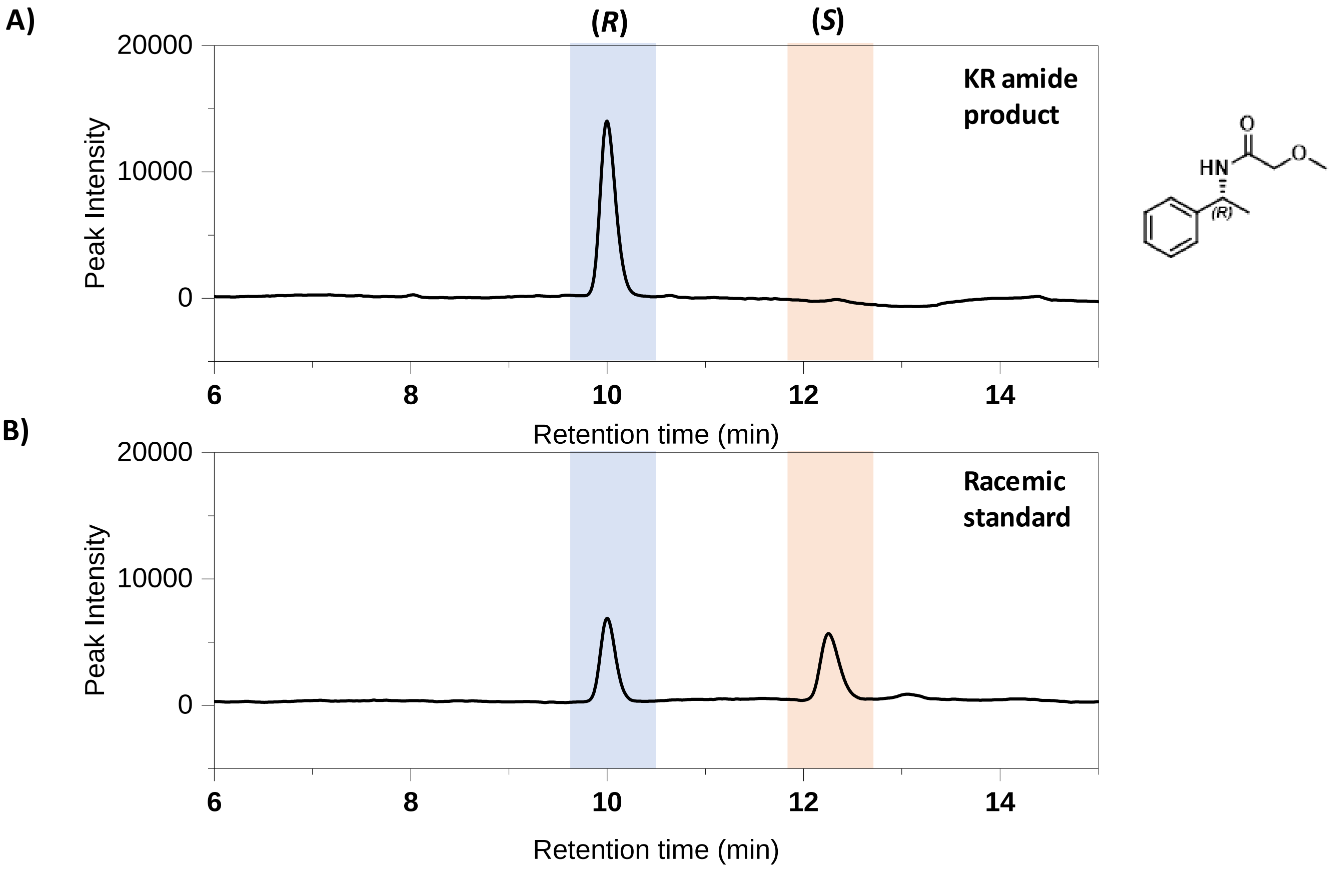

Figure S15. Chiral HPLC chromatograms for **A)** the amide product formed in the kinetic resolution of 1-phenylethylamine by CALB, (R)-2-Methoxy-N-(1-phenylethyl)acetamide (99.0% *ee*) and **B)** the racemic standard of 2-Methoxy-N-(1-phenylethyl)acetamide, synthesised chemically. t_R (*R*)-amide = 10.0 min (blue), t_R (*S*)-amide = 12.2 min (peach), HPLC method B.

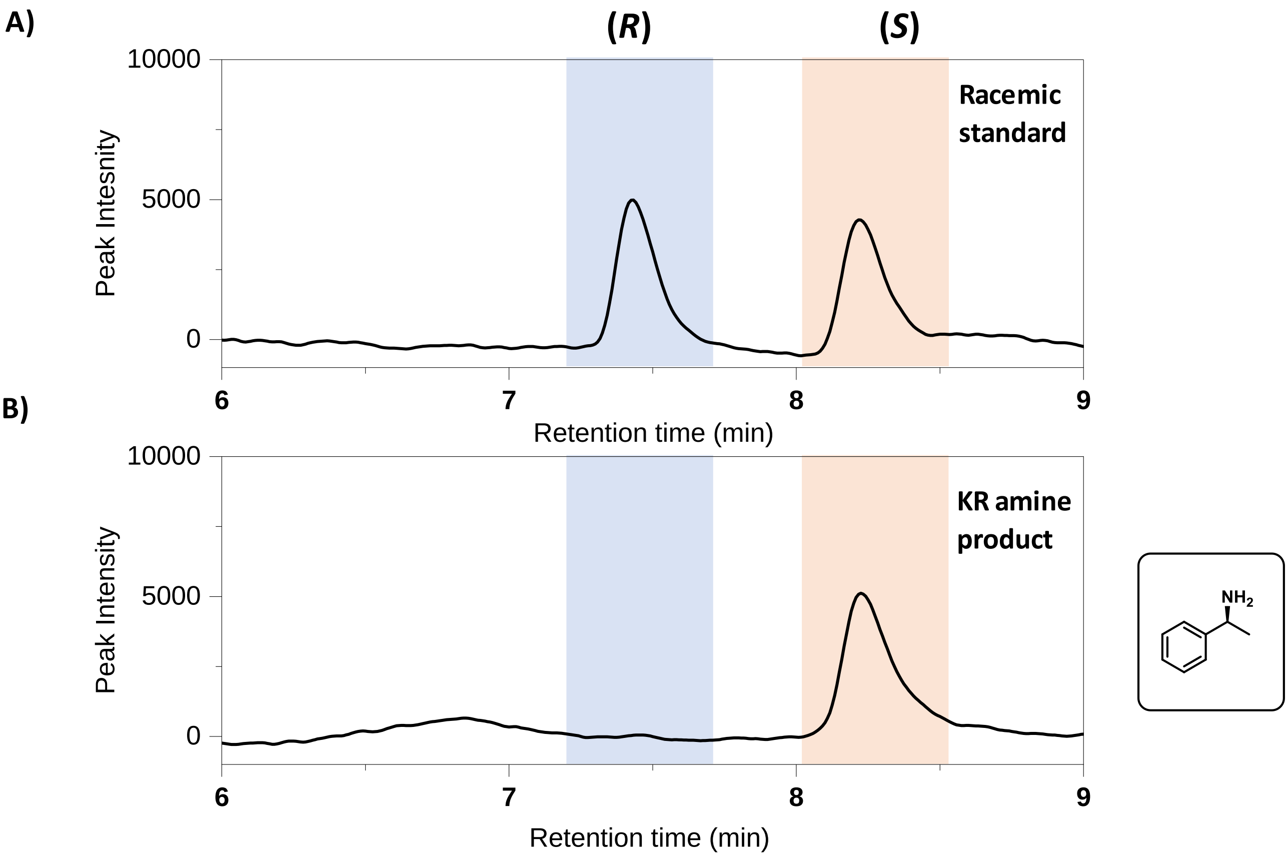

Figure S16. Chiral HPLC chromatograms for **A)** the amine product formed in the kinetic resolution of 1-phenylethylamine by CALB, (*S*)*-1-phenylethylamine* (99.6% *ee*) and **B)** the racemic standard of *1-phenylethylamine*, synthesised chemically. t_R (*R*)-amine = 7.4 min (blue), t_R (*S*)-amine = 8.2 min (peach), HPLC method B.

NMR spectra

(*R*)-2-Methoxy-N-(1-phenylethyl)acetamide

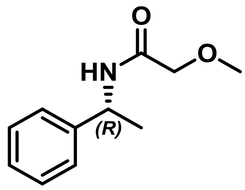

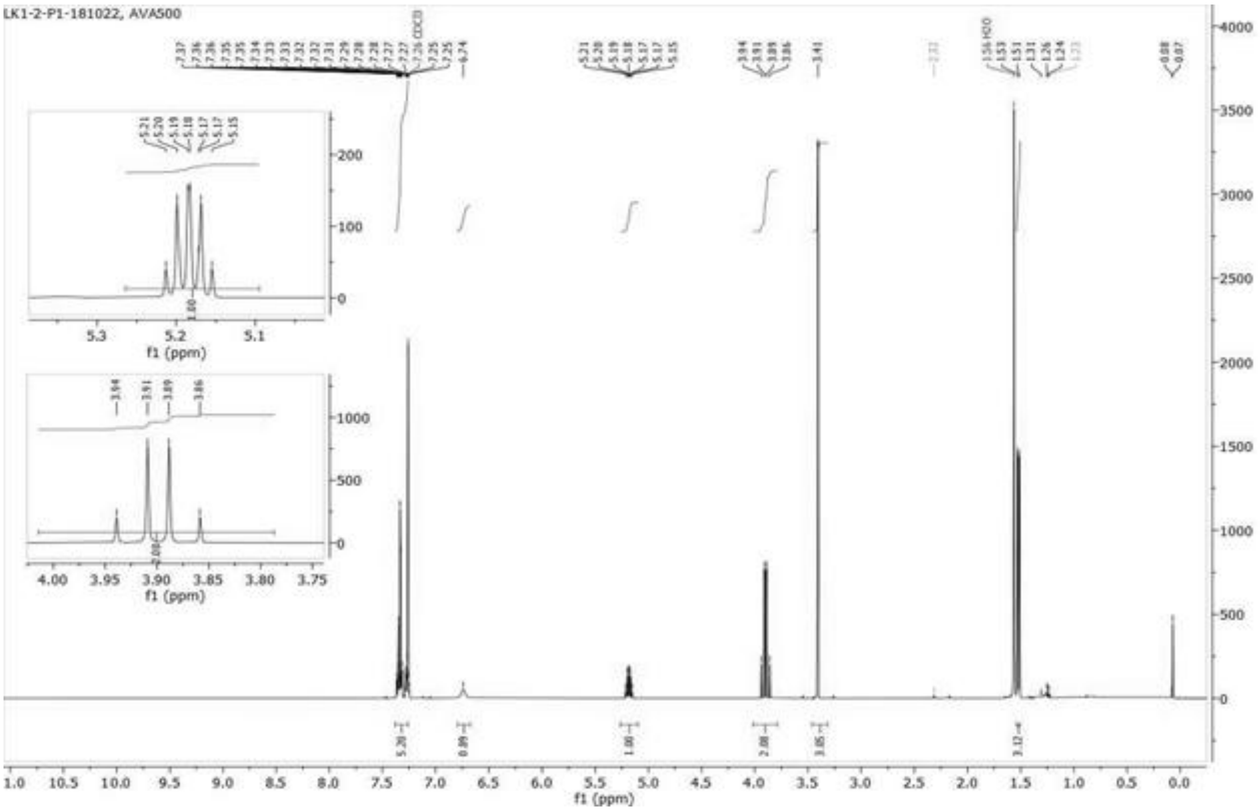

Figure S17. ^{1}H-NMR of (*R*)-2-Methoxy-N-(1-phenylethyl)acetamide in $CDCl_3$, 500 MHz.

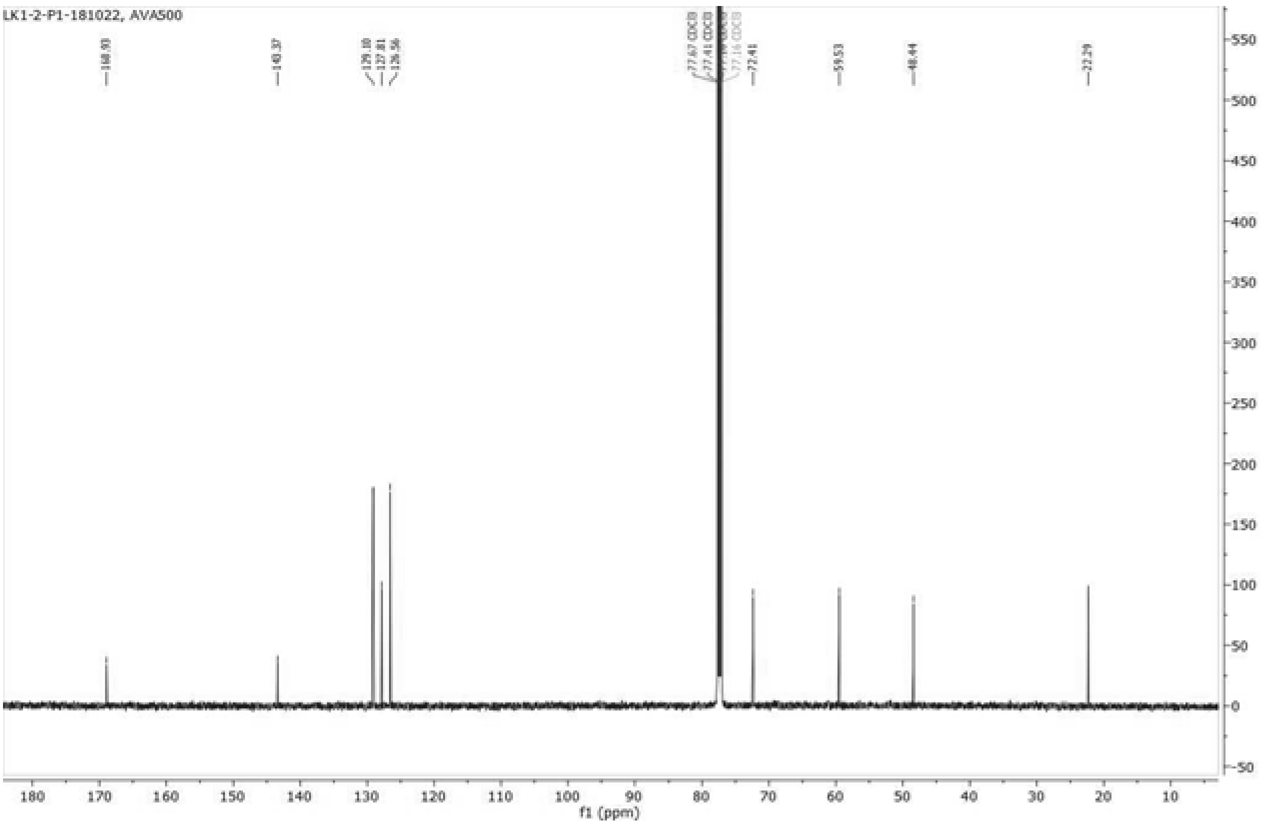

Figure S18. ^{13}C-NMR of (*R*)-2-Methoxy-N-(1-phenylethyl)acetamide in $CDCl_3$, 500 MHz.

(S)-1-phenylethylamine

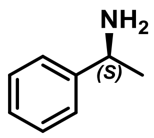

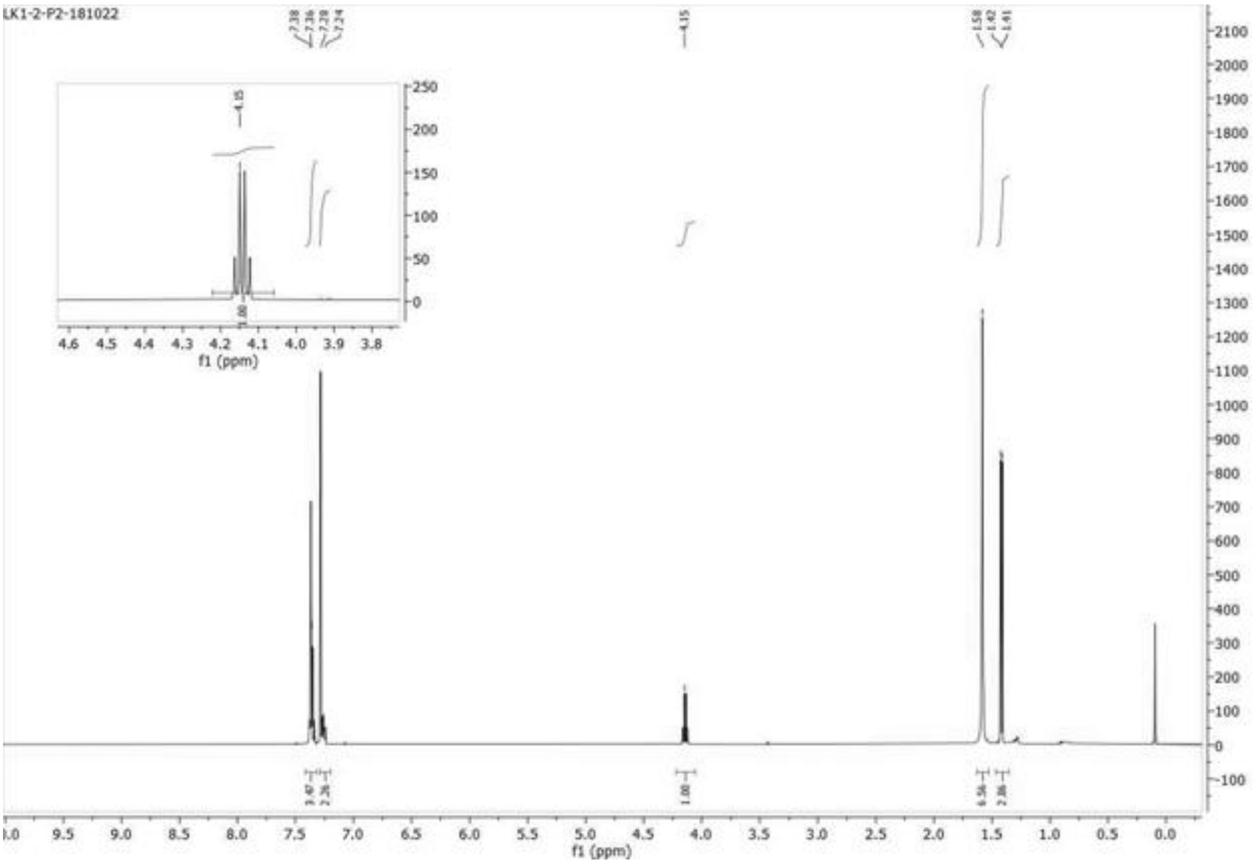

Figure S19. ^{1}H-NMR spectrum of (S)-1-phenylethylamine in $CDCl_3$, 500 MHz.

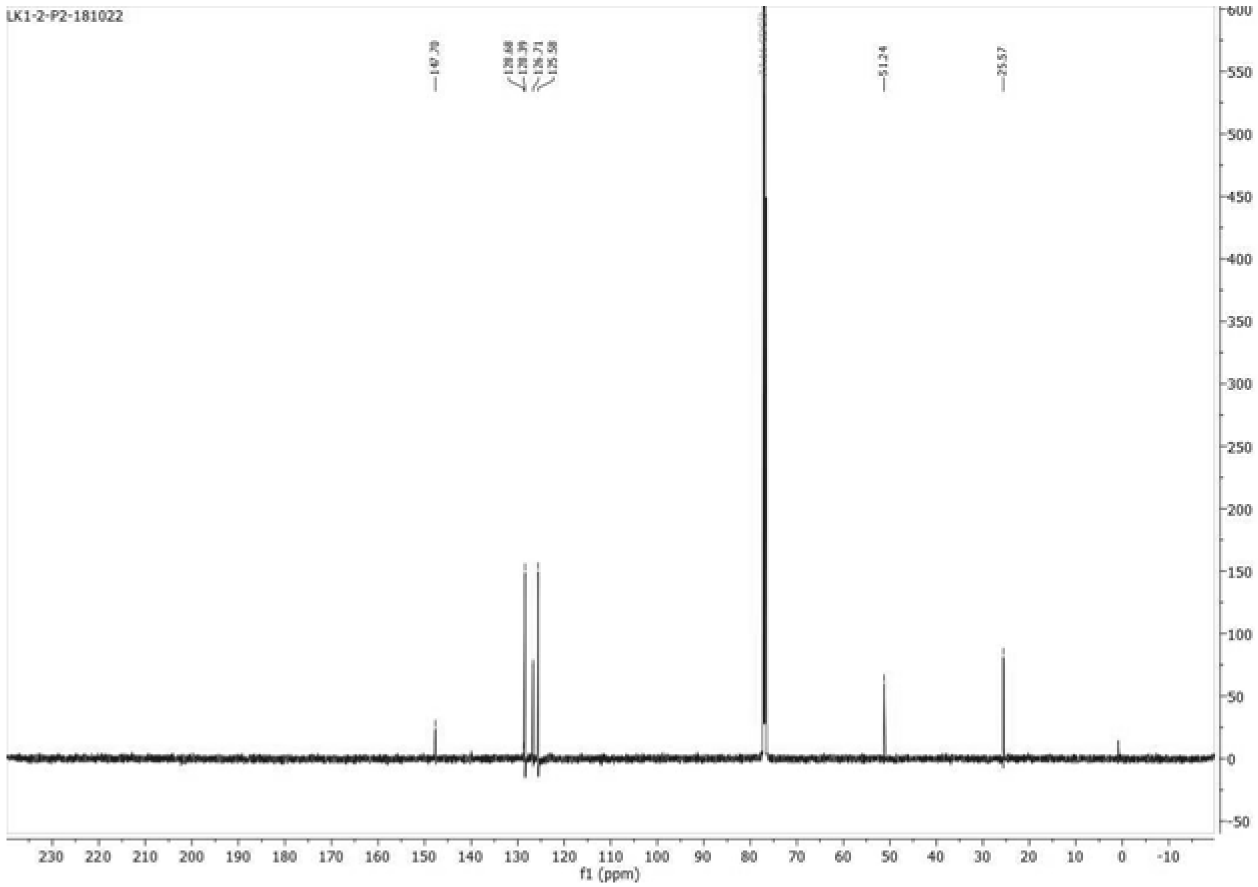

Figure S20. ^{13}C-NMR of (S)-1-phenylethylamine in $CDCl_3$, 500 MHz.

Phenoxy-2-propanamine

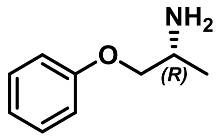

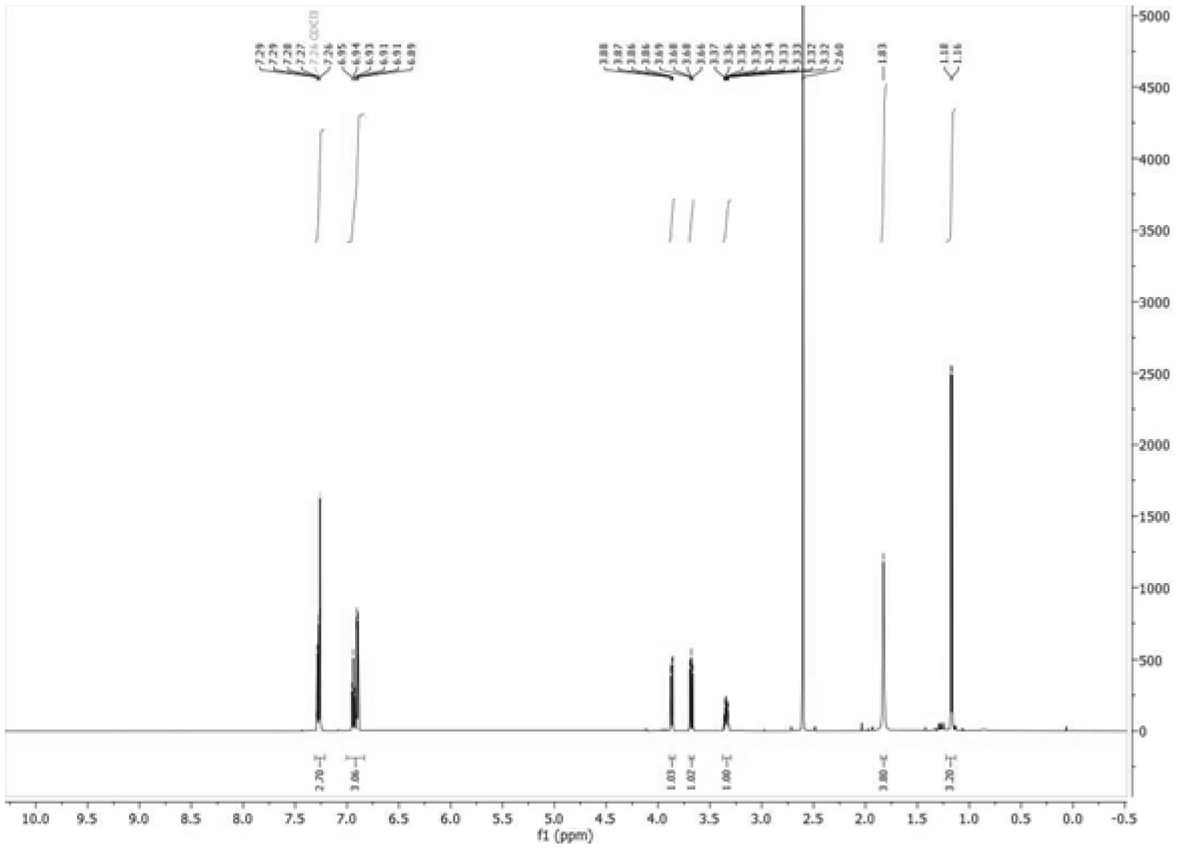

Figure S21. ^{1}H-NMR spectrum of phenoxy-2-propanamine formed by the transamination of phenoxy-2-propanone by ArRmut11-EMC7528.

Phenoxy-2-propanamine

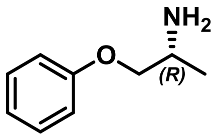

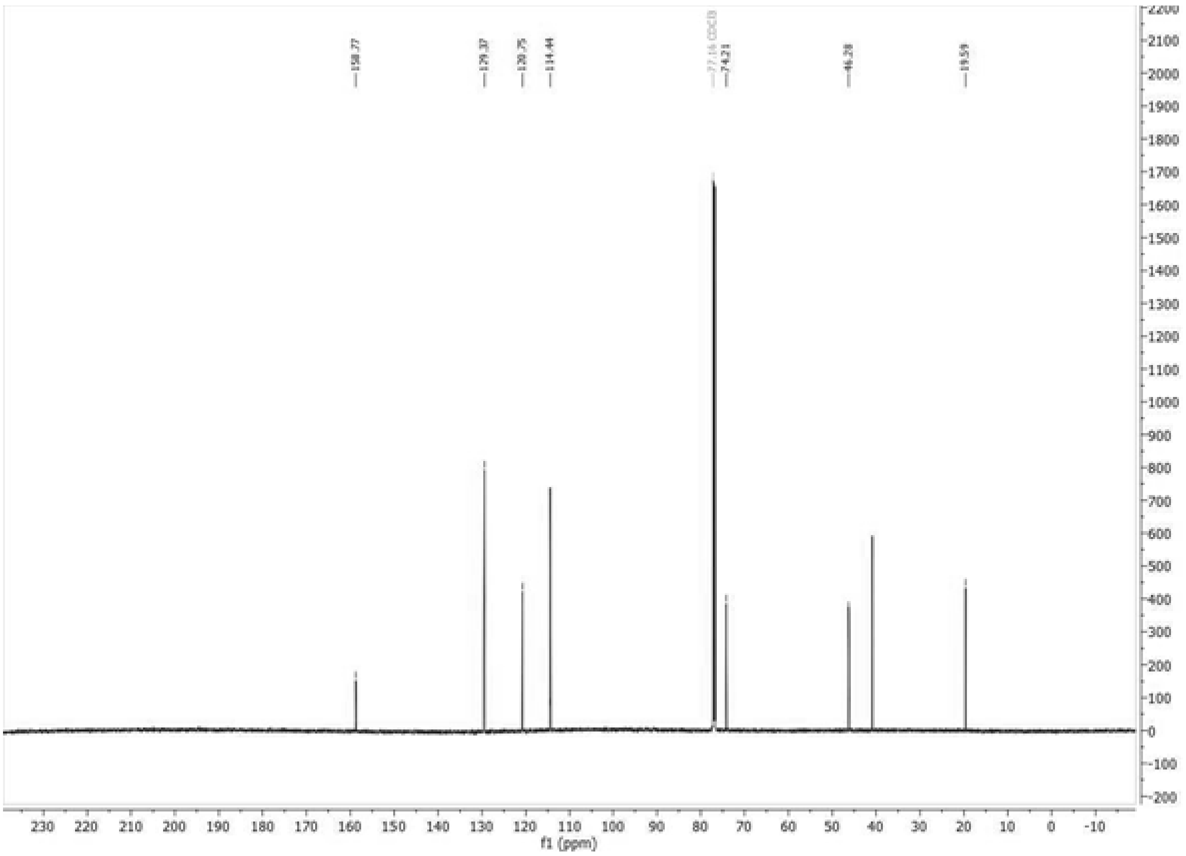

Figure S22. ^{13}C-NMR spectrum of purified phenoxy-2-propanamine formed by the transamination of phenoxy-2-propanone by ArRmut11-EMC7528.

Marfey's Reagent

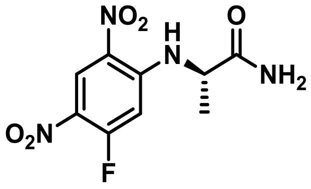

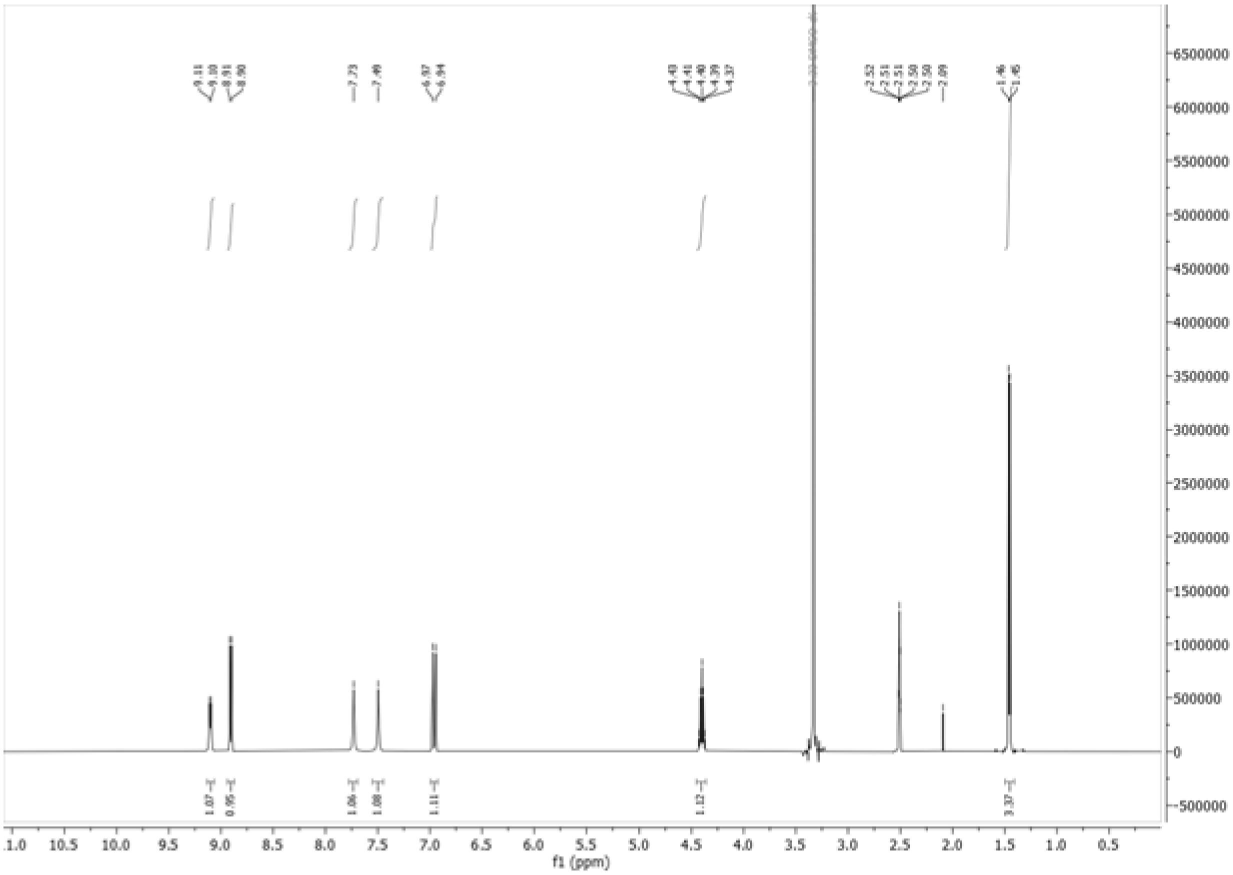

Figure S23. ^{1}H-NMR spectrum of Marfeys Reagent.

Marfeys Reagent

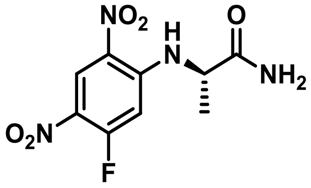

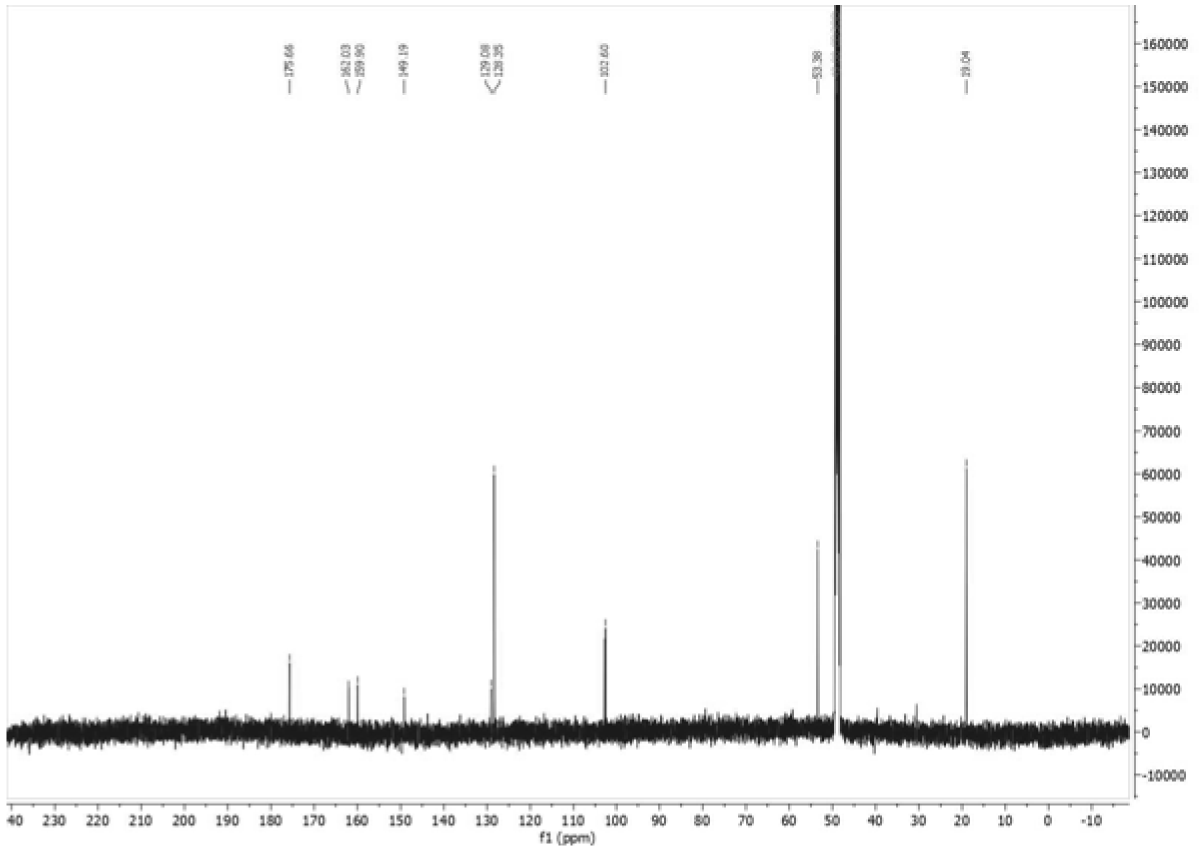

Figure S24. ^{13}C-NMR spectrum of purified Marfey's Reagent.

2-Methoxy-N-(1-phenoxy-2-propanyl)acetamide

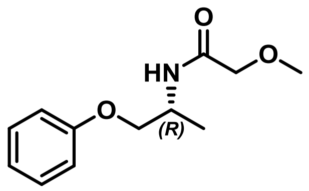

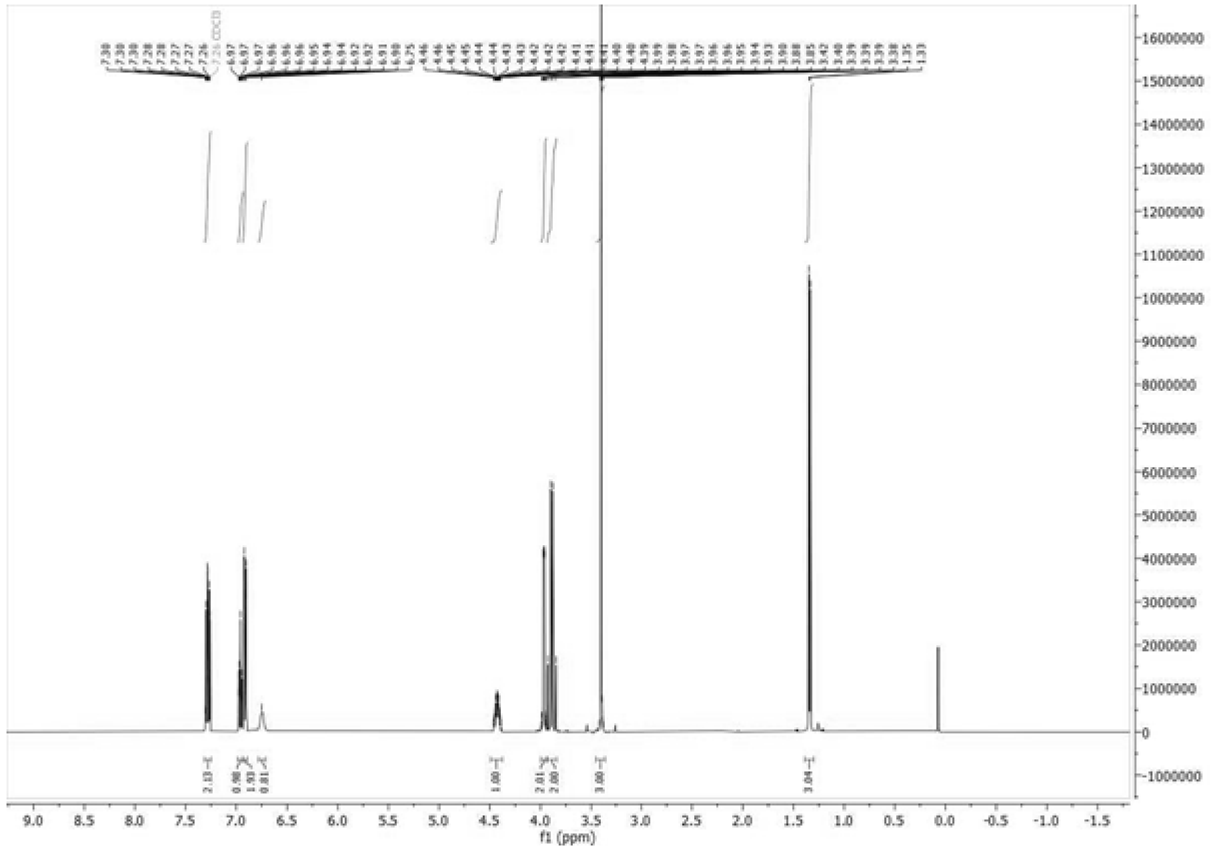

Figure S25. ^{1}H-NMR spectrum of cascade product, 2-methoxy-N(1-phenoxy-2-propanyl)acetamide.

2-Methoxy-N-(1-phenoxy-2-propanyl)acetamide

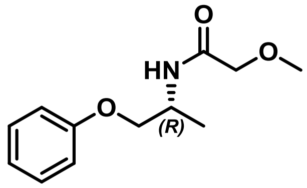

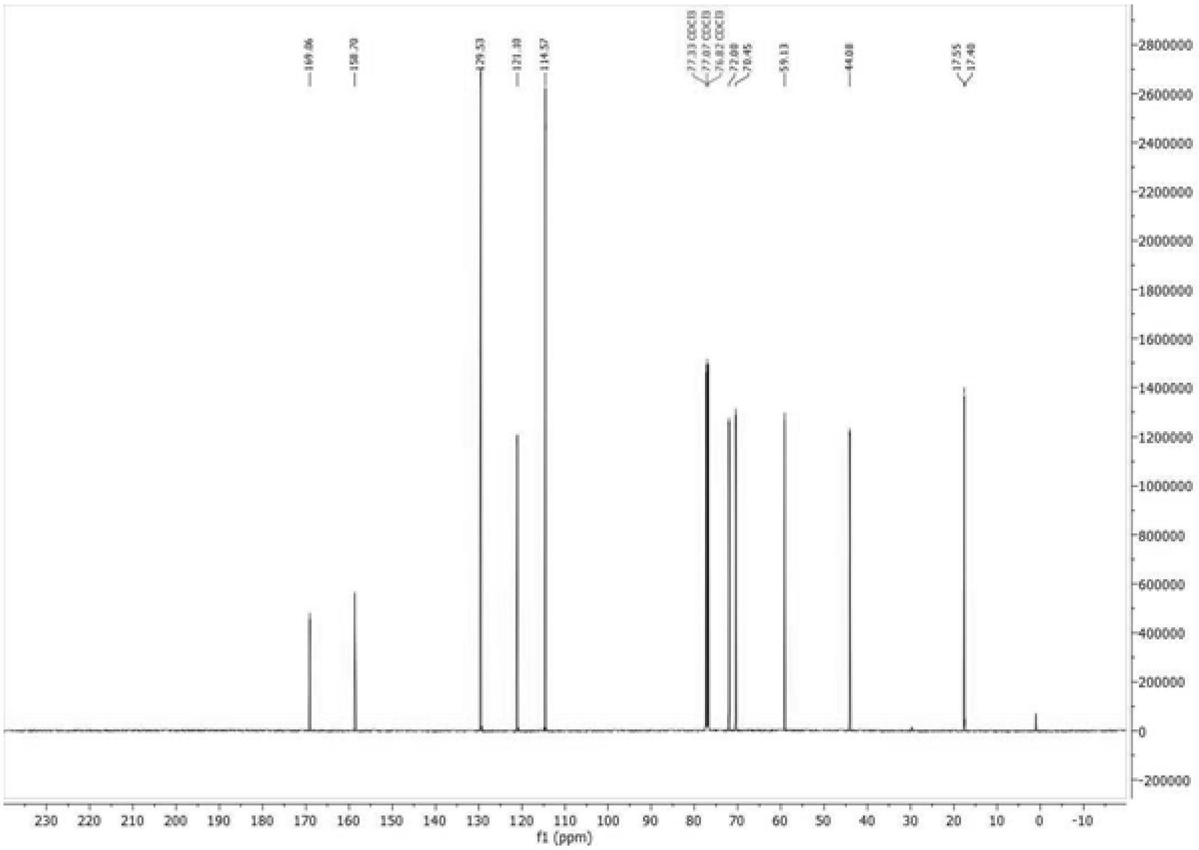

Figure S26. ^{13}C NMR spectrum of cascade product, 2-methoxy-N(1-phenoxy-2-propanyl)acetamide.

2-Methoxy-N-(1-phenoxy-2-propanyl)acetamide

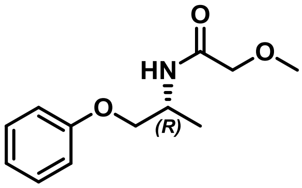

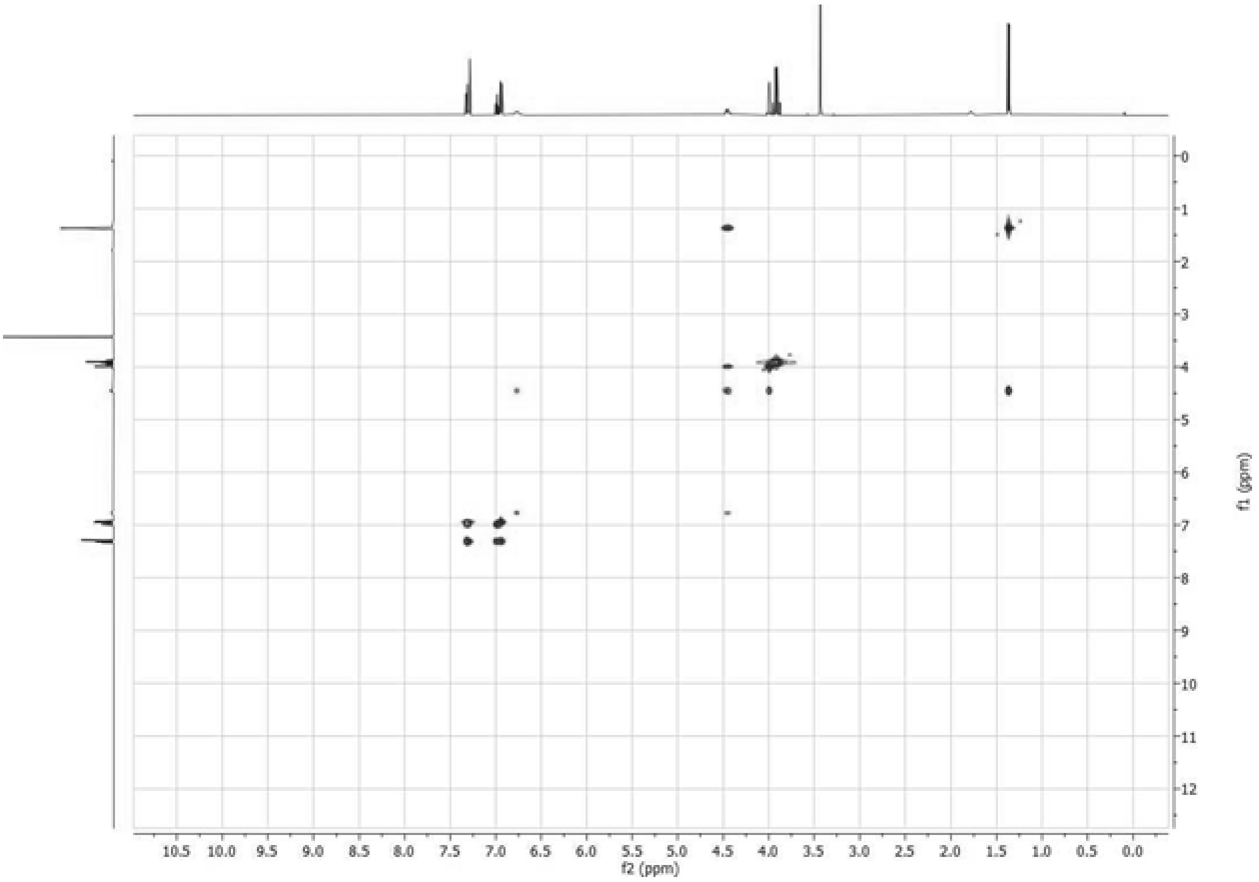

Figure S27. ^{1}H-^{1}H COSY NMR spectrum of cascade product, 2-methoxy-N(1-phenoxy-2-propanyl)acetamide.

HPLC chromatograms

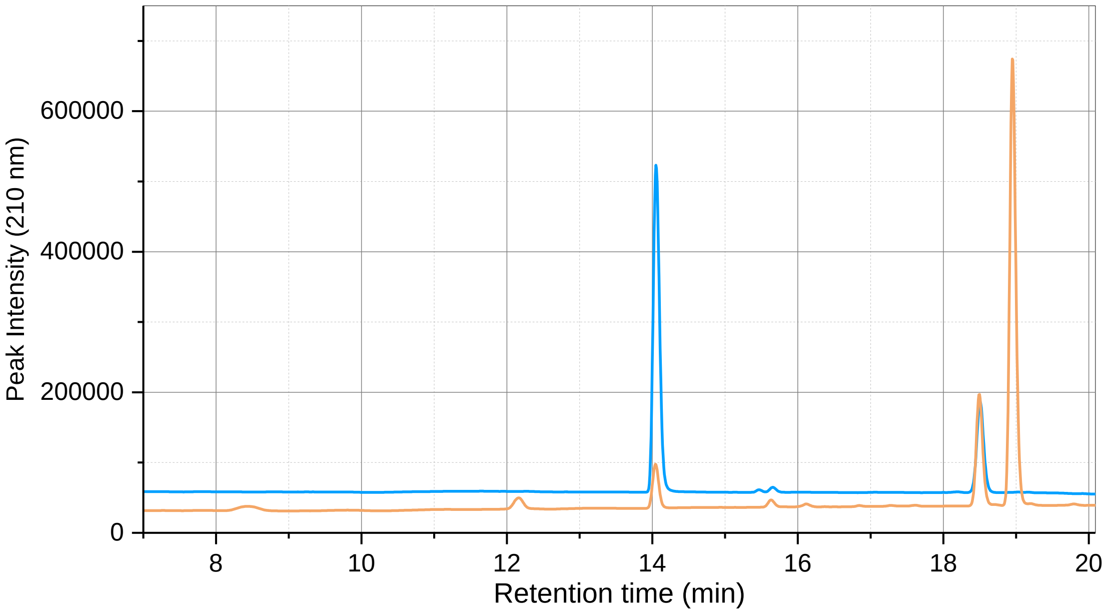

Figure S28. HPLC chromatograms for the cascade reaction. Reaction mixture after ArRmut11 step in blue and reaction mixture after CALB step in orange. Peak at 14.2 min corresponds to intermediate amine, phenoxy-2-propanamine. Peak at 18.5 min corresponds to ketone substrate, phenoxy-2-propanone. Peak at 19.0 min corresponds to amide product, 2-methoxy-N(1-phenoxy-2-propanyl)acetamide.

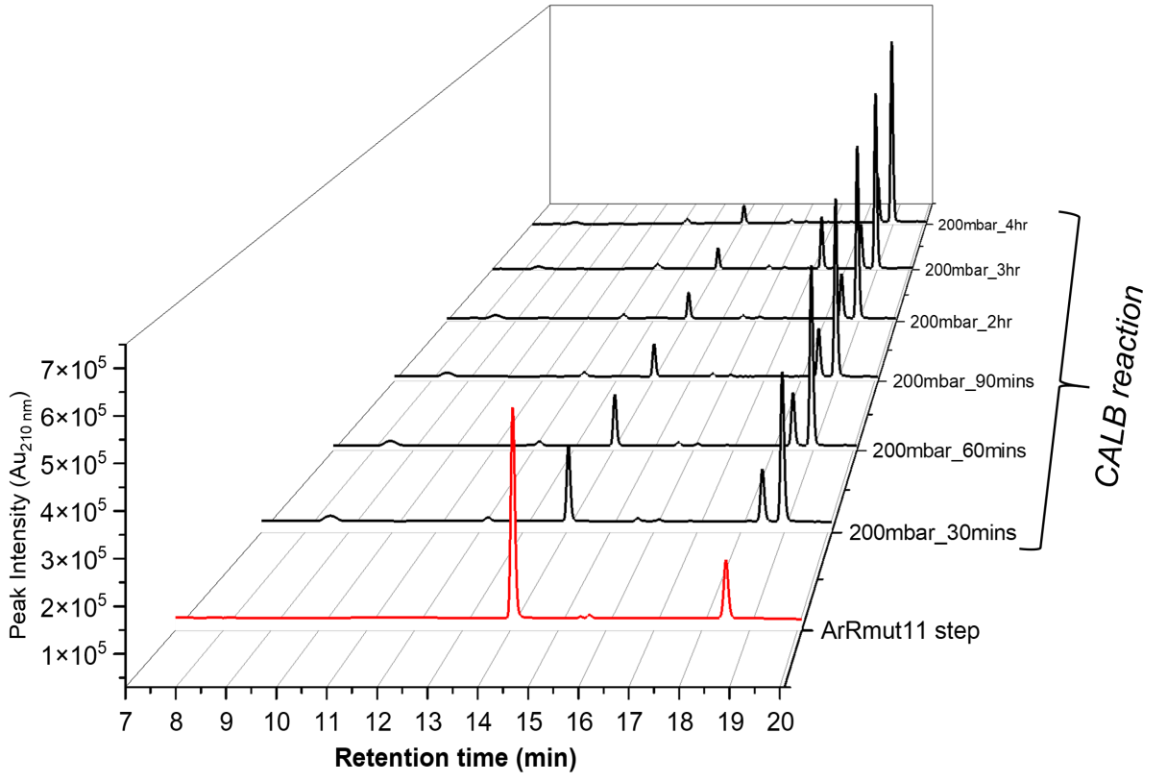

Figure S29. Stacked HPLC chromatograms from the reaction monitoring of the CALB step of the cascade reaction. The chromatogram in red corresponds to the reaction mixture at the end of the ArRmut11 step while the chromatograms in black correspond to time points taken during the CALB step under reduced pressure (200 mbar), showing the consumption of the intermediate amine and the formation of the amide product. Time points (30 mins to 4 hours) are detailed in the Z-axis labels. Peak at 14.2 min corresponds to intermediate amine, phenoxy-2-propanamine. Peak at 18.5 min corresponds to ketone substrate, phenoxy-2-propanone. Peak at 19.0 min corresponds to amide product, 2-methoxy-N(1-phenoxy-2-propanyl)acetamide.

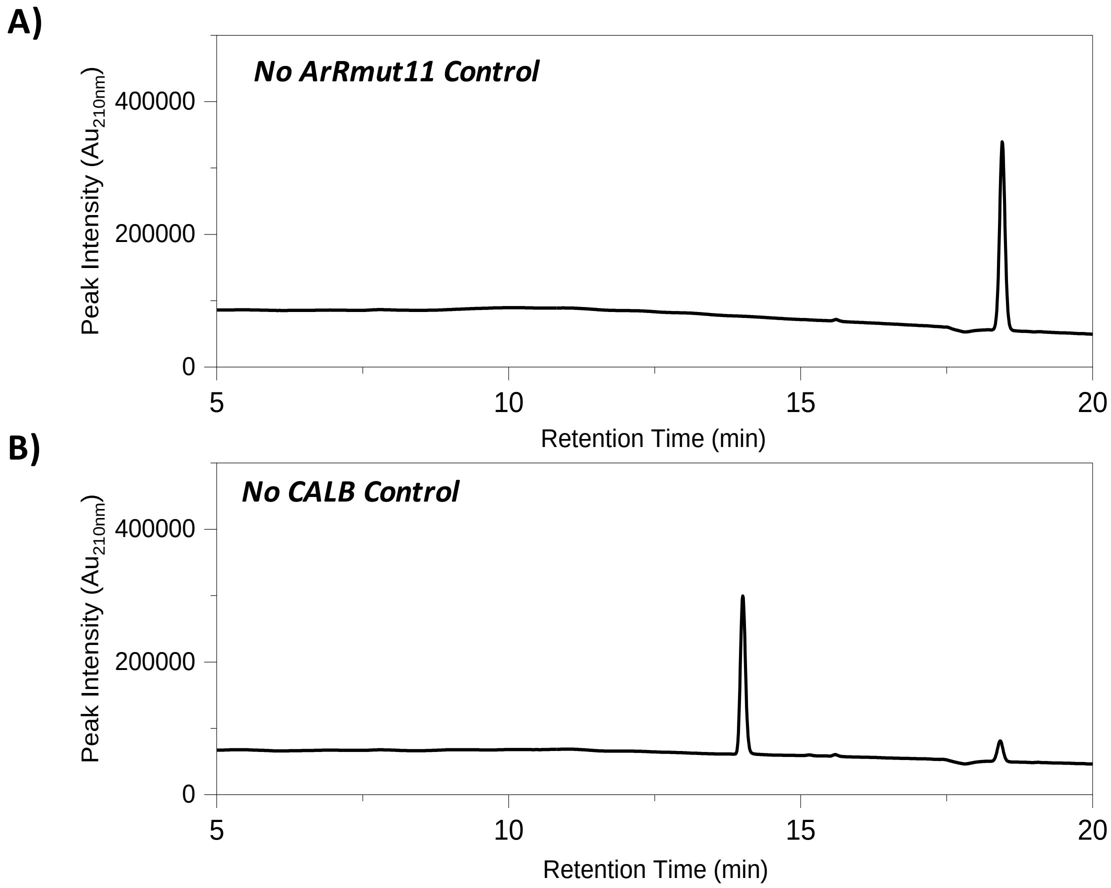

Figure S30. HPLC chromatograms for the no enzyme control reactions of the cascade. A) No ArRmut11 in the transamination reaction. B) No CALB in the second step of the cascade. Peak at 14.2 min corresponds to intermediate amine, phenoxy-2-propanamine. Peak at 18.5 min corresponds to ketone substrate, phenoxy-2-propanone. In the no CALB control, the reaction was subjected to the transamination reaction with ArRmut11-EMC7528, then methyl methoxyacetate was added but no CALB.

References

[1] C. A. McKenna, M. Štiblariková, I. De Silvestro, D. J. Campopiano, A. L. Lawrence, *Green Chem.* **2022**, *24*, 2010-2016.

[2] K. P. J. Gustafson, R. Lihammar, O. Verho, K. Engström, J.-E. Bäckvall, *J. Org. Chem.* **2014**, *79*, 3747-3751.

[3] M. J. Takle, B. J. Deadman, K. Hellgardt, J. Dickhaut, A. Wieja, K. K. M. Hii, *ACS Catal.* **2023**, *13*, 10541-10546.

[4] Y. Kita, M. Kuwabara, S. Yamadera, K. Kamata, M. Hara, *Chem. Sci.* **2020**, *11*, 9884-9890.

[5] K. P. J. Gustafson, T. Görbe, G. De Gonzalo-Calvo, N. Yuan, C. L. Schreiber, A. Shchukarev, C. W. Tai, I. Persson, X. Zou, J. E. Bäckvall, *Chem. Eur. J.* **2019**, *25*, 9174-9179.

[6] C. K. Savile, J. M. Janey, E. C. Mundorff, J. C. Moore, S. Tam, W. R. Jarvis, J. C. Colbeck, A. Krebber, F. J. Fleitz, J. Brands, P. N. Devine, G. W. Huisman, G. J. Hughes, *Science* **2010**, *329*, 305-309.

[7] D. Roura Padrosa, V. Marchini, F. Paradisi, *Bioinformatics* **2021**, *37*, 2761-2762.

[8] C. K. Prier, K. Camacho Soto, J. H. Forstater, N. Kuhl, J. T. Kuethe, W. L. Cheung-Lee, M. J. Di Maso, C. M. Eberle, S. T. Grosser, H.-I. Ho, E. Hoyt, A. Maguire, K. M. Maloney, A. Makarewicz, J. P. McMullen, J. C. Moore, G. S. Murphy, K. Narsimhan, W. Pan, N. R. Rivera, A. Saha-Shah, D. A. Thaisrivongs, D. Verma, A. Wyatt, D. Zewge, *ACS Catal.* **2023**, *13*, 7707-7714.

[9] R. M. Lanigan, V. Karaluka, M. T. Sabatini, P. Starkov, M. Badland, L. Boulton, T. D. Sheppard, *Chem. Commun.* **2016**, *52*, 8846-8849.

[10] D. Talwar, N. P. Salguero, C. M. Robertson, J. Xiao, *Chem. Eur. J.* **2014**, *20*, 245-252.

[11] J. Eberhardt, D. Santos-Martins, A. F. Tillack, S. Forli, *J. Chem. Inf. Model.* **2021**, *61*, 3891-3898.

[12] O. Trott, A. J. Olson, *J. Comput. Chem.* **2010**, *31*, 455-461.
